# Dual RNA-seq of maize and *H. seropedicae* ZAE94 association, in different doses of nitrate, reveals novel insights into Plant-PGPB-environment relationship

**DOI:** 10.3389/fpls.2024.1346523

**Published:** 2024-03-13

**Authors:** Aline Cardozo Rosman, Maria Clara de Oliveira Urquiaga, Flávia Thiebaut, Helkin Giovani Forero Ballesteros, Eduardo Alves Gamosa de Oliveira, Adriana Silva Hemerly

**Affiliations:** Laboratório de Biologia Molecular de Plantas, Instituto de Bioquímica Médica Leopoldo de Meis, Universidade Federal do Rio de Janeiro, Rio de Janeiro, RJ, Brazil

**Keywords:** *Zea mays* sp., beneficial bacteria, beneficial association, differentially expressed genes, transcriptome, hormone metabolism, cell wall metabolism, nitrogen metabolism

## Abstract

The interactions between plants, beneficial bacteria and their environment are profoundly shaped by various environmental factors, including light, temperature, water availability, and soil quality. Despite efforts to elucidate the molecular mechanisms involved in the association between plants and beneficial bacteria, like Plant Growth-Promoting Bacteria (PGPB), with many studies focusing on the transcriptional reprogramming in the plant, there is no report on the modulation of genetic controls from both plant and associated bacteria standpoints, in response to environment. The main goal of this study was to investigate the relationship between plant-bacteria-environment signaling, using as a model maize plants inoculated with *H. seropedicae* ZAE94 and cultivated with different doses of N (0.3 and 3 mM). For this purpose, we performed rRNA-depleted RNA-seq to determine the global gene expression of both maize roots and associated *H. seropedicae* ZAE94. Our results revealed a differential modulation of maize nitrogen metabolism, phytohormone and cell wall responses when associated with *H. seropedicae* ZAE94 at different N concentrations. In parallel, a modulation of the bacterial metabolism could be observed, by regulating genes involved in transport, secretion system, cell mobility, oxidoreductases, and chemotaxis, when bacteria were associated with maize roots and cultivated at different doses of N. The molecular and phenotypic data of maize plantlets suggested that different doses of N fertilization differentially regulated the beneficial effects of bacterial inoculation, as higher doses (3 mM) favored shoot elongation and lower doses (0.3 mM) favored increase in plant biomass. Our results provide a valuable integrated overview of differentially expressed genes in both maize and associated *H. seropedicae* ZAE94 in response to different N availability, revealing new insights into pathways involved in grass-PGPB associations.

## 1 Introduction

The world production and productivity of maize (*Zea mays* L.) has doubled in the last two decades, resulting in a production of 1.2 billion tons of grains in 2022 (FAOSTAT, 2024a). This significant increase in productivity is mainly attributed to chemical inputs, crop management and genetic improvement (Ladha et al., 2016; Mueller et al., 2019). Agricultural use of inorganic fertilizers in 2021 was just above 195 million tons of nutrients, of which 56% was nitrogen (N) (FAOSTAT, 2024b). However, recent results have demonstrated that the indiscriminate use of nitrogenous chemical fertilizers has caused serious environmental problems (Keeler et al., 2016; Mateo-Sagasta et al., 2017; Kanter, 2018), raising severe concerns about the dependence of modern agriculture on these inputs. In addition to the negative environmental impact, N fertilizers also account for about 20% of maize production costs (Richetti and Ceccon, 2020).

Although maize is highly dependent on N fertilization, N use efficiency (NUE) is estimated to be less than 50% (Mueller et al., 2019). According to Sheoran et al. (2021), a promising alternative strategy to reduce dependence on N fertilization in plant cultivation is the inoculation with plant growth-promoting bacteria (PGPB). These bacteria can enhance NUE in plants through various mechanisms. Nitrogen-fixing PGPB can directly contribute N to plants, while PGPB in general can benefit plants by improving assimilation of nitrogen, solubilizing nutrients like phosphate, producing hormones, enhancing root architecture, providing biocontrol against pests and diseases, and improving stress tolerance (Di Benedetto et al., 2017; Zeffa et al., 2019; Pankievicz et al., 2021; Gaspareto et al., 2023). These actions collectively lead to increased NUE by enhancing nutrient uptake and overall plant health, reducing the reliance on synthetic N fertilizers.

Among the genera of PGPB that establish efficient associations with different crop species, *Herbaspirillum* stands out as a model to study the processes of bacterial recognition, colonization, and growth promotion in grasses, being recommended as maize inoculant (Reis et al., 2010). *Herbaspirillum seropedicae* is an endophytic β-proteobacteria found naturally associating with several economically important grasses such as sugarcane, wheat, rice, and maize, increasing crop productivity (Baldani et al., 1986; Olivares et al., 1996; Gyaneshwar et al., 2002; Monteiro et al., 2008). Despite being frequently found in the rhizosphere, endophytic colonization by *H. seropedicae* is based on the attachment of the bacteria to root surfaces and the subsequent colonization of the emergence points of lateral roots (Monteiro et al., 2012). In particular, *H. seropedicae* strain ZAE94, originally isolated from rice roots, has been extensively tested in maize showing great potential as PGPB (Alves et al., 2015; Breda et al., 2019; Alves et al., 2021).

The inoculation of *H. seropedicae* in maize can alter the N metabolism and plant development, increasing length and volume of roots, promoting leaf growth, as well as the accumulation of N, P and K in the aerial part, and ensuring better stress tolerance (Alves et al., 2015; Breda et al., 2016; Curá et al., 2017; Hardoim et al., 2019; Alves et al., 2021; Nunes et al., 2021). Furthermore, foliar inoculation of *H. seropedicae* in maize can also increase grain yield by up to 65% (Canellas et al., 2013). At gene expression level, a previous work by our research group showed that the expression pattern of N metabolism genes was modulated in whole maize seedlings inoculated with *Azospirillum brasilense* Sp245 and *H. seropedicae* HRC54, suggesting that some responses are common to both beneficial bacteria, while others are specific for each bacterial species (Hardoim et al., 2019). In addition to modulation of genes related to N, the expression of gene clusters that might be related to plant-microbe interaction, cell wall, hormones and of several transcription factors allow the bacteria to recognize plant signals and modulate plant gene expression for endophytic colonization and plant growth promotion (de Carvalho et al., 2011; Thiebaut et al., 2014; de Azevedo et al., 2019; Hardoim et al., 2019).

The interactions between plants, beneficial bacteria and their environment are profoundly shaped by various environmental factors, including light, temperature, water availability, and soil quality (Vargas et al., 2014; Li et al., 2019; Thiebaut et al., 2022; Leisner et al., 2023). These factors dictate a plant’s ability to thrive, affecting growth, resilience, and overall health. Simultaneously, the environment plays a pivotal role in determining the efficacy of PGPB. PGPB thrive in specific environmental conditions that support their growth and activity, and their ability to enhance plant growth is intricately tied to the soil quality and nutrient availability in the ecosystem (Berthrong et al., 2014; Zhou et al., 2015; Wang et al., 2016a; Ding et al., 2017; Wang et al., 2017; Fan et al., 2019; Zhou et al., 2021). When plants, PGPB and the environment function synergistically, they create a harmonious ecosystem where plants are well-adapted to their surroundings, and PGPB optimize nutrient uptake and bolster defense mechanisms. This integrated relationship between plant-bacteria-environment not only benefits agriculture through increased yields and reduced chemical inputs but also supports biodiversity and environmental health. Recognizing and managing these intricate relationships is crucial for sustainable agriculture and the preservation of natural ecosystems.

Despite efforts to elucidate the molecular mechanisms involved in the interaction between maize and PGPB, with many studies focusing on the transcriptional reprogramming in maize (Alves et al., 2015; Breda et al., 2016; Curá et al., 2017; Hardoim et al., 2019; Alves et al., 2021; Nunes et al., 2021) or *H. seropedicae* ZAE94 (Tadra-Sfeir et al., 2015; Balsanelli et al., 2016; Pankievicz et al., 2016; Brusamarello-Santos et al., 2019; Grillo-Puertas et al., 2021; Pessoa et al., 2021), there is no report on the modulation of genetic controls in response to plant-bacteria-environment interactions, from both plant and associated bacteria standpoints. In the present work, we investigated the integration between plant-bacteria-environment signaling, based on the association of maize plants inoculated with *H. seropedicae* ZAE94 and cultivated with different doses of N. The investigation used different approaches, such as: (i) phenotypic analysis of plant growth; (ii) quantification of bacterial colonization; (iii) ribosomal RNA-depleted (rRNA-depleted) RNA sequencing (RNA-seq) to determine the global gene expression of both maize and *H. seropedicae* ZAE94, when associated and cultivated under different doses of N; (iv) validation of gene expression profiles by qRT-PCR. Our results provide the first integrated overview of differentially expressed genes in both maize and associated *H. seropedicae* ZAE94 in response to different N availability, revealing new insights into pathways involved in grass-PGPB associations, potentially offering sustainable alternatives to reduce chemical fertilizers dependence in agriculture.

## 2 Materials and methods

### 2.1 Plant material and PGPB inoculation

**Figure 1:**
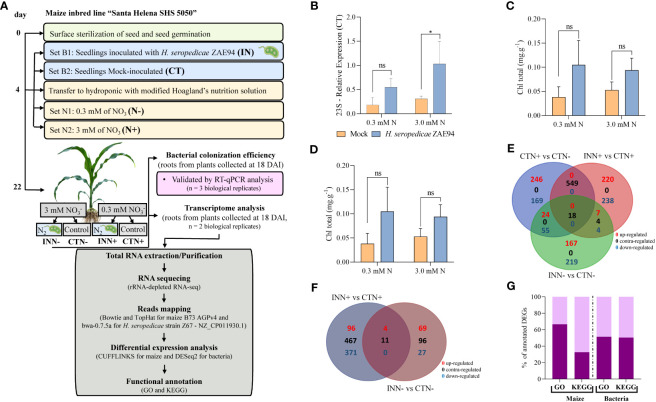
Nitrogen assay of maize colonized with beneficial diazotrophic bacteria. **(A)** Experimental design for the transcriptome analyses of maize inbred line Santa Helena SHS 5050 with diazotrophic bacteria *H. seropedicae* ZAE94 (IN) or mock-inoculated (CT). Four-day-old maize seedlings were inoculated with or without bacteria and transferred to a hydroponic system with modified Hoagland’s nutrition solution containing 0.3 mM (N-) or 3 mM (N+) of nitrate. Maize root samples were collected at eighteen days after inoculation (DAI) for transcriptome analysis. **(B)** qRT-PCR quantification of maize colonization by *H. seropedicae* 18 DAI. Bacterial *23S rRNA* levels are presented relative to *28S* levels in each sample. Bars represent mean ± standard deviation of the relative mRNA expression in three biological replicates and each biological replicate analyzed with three technical replicates. Means with asterisk are significantly different at 5% level of confidence (One-way ANOVA test with Tukey’s post-test) **(C, D)** Total chlorophyll (Chl) content. Total carotenoids (Car) content, respectively. Bars represent mean ± standard deviation of three biological replicates. Means with asterisk are significantly different at 5% level of confidence and means with ns were not significant (One-way ANOVA test with Tukey’s post-test) **(E, F)** Venn diagrams of DEGs in maize and in the bacteria, respectively. **(G)** Functional annotation of DEGs in GO and KEGG.

For RNA-seq library construction, the maize (*Zea mays* L.) inbred line Santa Helena SHS 5050, which is a triple hybrid responsive to *H. seropedicae* ZAE94 (BR 11417) (Alves et al., 2015, 2021), was used. The hydroponic experiment set up and performed analyses are described in **Figure 1** and **Table 1**. Briefly, maize seeds were pre-washed with sterile ddH_2_O and then surface-sterilized using a solution of 0.5% (v/v) sodium hypochlorite and 0.01% (v/v) Tween 20 for 5 minutes under constant smooth agitation. After that, the seeds were washed 3 times (5 minutes each) with sterile phosphate buffer (KH_2_PO_4_, 50 mmol L^−1^ pH 7.0) under the same conditions as described above. Seeds were sown into phenolic foam and incubated in a growth chamber for 4 days at 30°C for germination and initial growth. After the first growth period, the seedlings were inoculated with 10^9^ cells mL^-1^ for 1 hour and then transferred to 5 L hydroponic containers where the experiment was performed. Briefly, for inoculation, the strain ZAE94 was used after it had been grown in DYGS medium (2 g/L malic acid; 2 g/L glucose; 1.5 g/L bacteriological peptone; 2 g/L yeast extract; 0.5 g/L K_2_HPO_4_; 0.5 g/L MgSO_4_.7H_2_O and 1.5 g/L glutamic acid, pH 6.0). Control seedlings were mock-inoculated with sterile DYGS. Each hydroponic container received 5 L modified half-strength Hoagland nutrient solution (Hoagland and Arnon, 1950) (**Supplementary Table S1**) and was kept under constant aeration. Two N concentrations (0.3 and 3 mM) were set up as low and high concentrations, respectively, and were applied after 6 days of acclimatization period. The experiment was performed in blocks in a completely randomized fashion, with five biological replicates for each treatment, and the plants were harvested 18 days after inoculation (DAI). Three biological replicates were used for the phenotypic analysis of the experiment and for molecular analyses to validate the RNA-seq experiment. Two biological replicates were sent for RNA sequencing.

**Table 1:** Abbreviations used for the sample types and dataset comparisons.

Conditions	Description of experimental conditions
CTN+	Mock-inoculated plants cultivated in 3 mM of Nitrate
CTN-	Mock-inoculated plants cultivated in 0.3 mM of Nitrate
INN+	Inoculated plants cultivated in 3 mM of Nitrate
INN-	Inoculated plants cultivated in 0.3 mM of Nitrate
Dataset comparisons (plant)	Dataset comparisons (bacteria)
CTN+ vs CTN-	–
INN+ vs CTN+	INN+ vs CTN+
INN- vs CTN-	INN- vs CTN-

In order to phenotypically and molecularly evaluate the responses of maize to inoculation under different doses of N, an experiment very similar to the one performed for RNA-seq library construction was carried out, using 10 biological replicates for phenotype analysis and three biological replicates for molecular analysis. Maize seeds were superficially sterilized by immersion in a disinfestation solution (0.5% NaOCl and 0.01% Tween 20) under agitation at 65 rpm, for 5 min at 30°C. Then, three washes of 5 min each were performed with a phosphate buffer solution (50 mM; pH 7), under the same conditions. Subsequently, the seeds were pre-germinated on germitest paper in growth chambers at 26/27°C with a photoperiod of 16/8 hours, for 4 days. After this period, maize seedlings were selected, choosing those with a root length of approximately 5 cm. The inoculant was prepared as in the prior experiment, and maize seedlings were inoculated with 10^9^ cells mL^−1^ for 1 hour. Following inoculation, the seedlings were transferred to test tubes containing 25 mL of modified Hoagland nutrient solution with 1.5 mM of N concentration. After a 6-days acclimatization period, the plants were transferred to modified Hoagland solution at 1/2 ionic force containing two different concentrations of N (0.3 and 3 mM), using Mg(NO_3_)_2_, (NH_4_)_2_SO_4_ and Ca(NO_3_)_2_ as a source of N (Dias et al., 2021). The plants were harvested 18 DAI.

### 2.2 Phenotypic analyses of maize plants

After separating the plants into shoots and roots, both parts were initially weighed in order to obtain fresh matter values. In addition, shoot length was measured from the end that continued the root to the end of the longest leaf. Subsequently, each shoot and root replica was placed in paper bags and left in an oven at 50°C for three days, when they were weighed again to obtain dry matter values. The values of shoot length and fresh and dry matter obtained were statistically analyzed using GraphPad Prism software version 8.0.0. Statistical analysis was performed using the Two-way ANOVA test with Tukey’s post-test. Statistical significance was defined in all cases as p-value < 0.05.

For analyses of root development, the harvested roots were immersed in 70% ethanol for 7 days. Then, roots were laid out in an acrylic container (30 × 40 cm), with water at an approximate depth of 1 cm, and placed onto the scanner. They were scanned and characterized by image analyses using WinRHIZO Pro® software (Regent Instruments, QC, Quebec, Canada) coupled to an Epson Expression 11000XL LA2400 image scanner, as described in previous works (Bauhus and Messier, 1999; Bouma et al., 2000; Dias et al., 2021). In total, 10 plants per treatment were analyzed. The surface area (m^2^) of the roots was evaluated. All analyzes used the ANOVA test with Tukey’s post-test, with a bilateral analysis. Statistical significance was defined as p-value < 0.05.

### 2.3 Photosynthetic pigments

Chlorophyll a (chl a [12.74 A665 – 3.62 A649]), chlorophyll b (chl b [25.06 A649 – 6.5 A665]), total chlorophyll (total chl [chl a + chl b]) and total carotenoid (total car [1000 A480 -1.29 Ca – 53.78 Cb)/220]) contents were determined in the complete third fully expanded leaves. The leaves were immersed in 5 mL of dimethyl sulfoxide (DMSO) and then incubated in the dark for 72 hours. Pigment concentrations were quantified using the spectrophotometric method at wavelengths of 649 and 665 nm for chlorophyll a, b and 480 nm for carotenoids. The pigment concentrations were determined as proposed by Wellburn (1994). Statistical analysis was based on the Two-way ANOVA test with Tukey’s post-test. Statistical significance was defined in all cases as p-value < 0.05.

### 2.4 RNA extraction and RNA-seq library construction

For all molecular analyses, plant roots were collected at 18 DAI and flash-frozen in liquid nitrogen. Samples of each treatment, i.e. maize seedlings inoculated with *H. seropedicae* ZAE94 and their respective mock-inoculated seedlings, were used for Illumina sequencing (**Table 1**). In addition, these samples were used for plant or bacteria transcriptome expression and bacterial colonization qRT-PCR validations. Total RNA was isolated from root plants using Trizol home-made as described by Valach (2014). RNA concentration was measured with a Nano-Drop™ 2000c spectrophotometer (Thermo Scientific, USA), and integrity was evaluated with an Agilent-2100 Bioanalyzer (Agilent Technologies; RNA 6000 NanoChips). Plant samples with a 10 ug total RNA were sent to Fasteris Life Sciences SA (Plan-les-Ouates, Switzerland) for RNA-seq library constructions and subsequent sequencing by Illumina technology. To prepare the eight libraries two protocols were combined. Total RNA samples were depleted using the module for rRNA depletion from the Illumina TruSeq Stranded total RNA Library Prep kit Plant. Depleted RNA was then used as input for library preparation with the Ovation Complete Prokaryotic RNA-seq DR Multiplex System 1-8 from Nugen. Briefly, cDNA sequencing library was prepared from the rRNA-depleted using the mRNA-seq HiSeq SBS Kit V4 (Illumina, San Diego, CA). Sequencing of paired-end RNA-seq libraries was performed using an Illumina HiSeq 2500 instrument at Fasteris Life Sciences SA (Plan-les-Ouates, Switzerland), following the manufacturer’s protocol. To ensure the reliability of our molecular analyses, we prioritized sequencing quality in this study. More than 93% of bases had a base quality ≥Q30, and the real-time error rate of spiked PhiX was 0.13%, indicating high sequencing quality. The datasets generated can be found at SRA/NCBI https://www.ncbi.nlm.nih.gov/sra/PRJNA1047090.

### 2.5 Transcriptome analysis

Sequence and primary analysis of the Illumina RNA-seq libraries of maize roots generated 495 million paired-end reads (see **Supplementary Table S2**). High quality reads for each sample were analyzed using the “Tuxedo Suite” package (Trapnell et al., 2012) against the reference maize B73 genome (Emsenbl AGPv4, from iGenome database). Briefly, the alignment of reads was performed with Bowtie and Tophat v2.0.9 (Kim et al., 2013). The transcript abundance, estimated with Cufflinks v2.1.1 (Trapnell et al., 2012), was reported as Fragments Per Kilobase of exon per Million fragments mapped (FPKM), serving as a normalized measure. The entire process, including normalization and comparison, was conducted using Cufflinks v2.1.1 software (Trapnell et al., 2012). Quality filtered RNA-seq reads were also mapped to reference genome sequences of *H. seropedicae* Z67 (NZ_CP011930.1) with Burrows-Wheeler Alignment (BWA) Tool v0.7.5a (Li and Durbin, 2010). The quantification was performed using bedtools, and for normalization and comparison, DESeq2 was used (Anders and Huber, 2010). The FPKM values from maize plants inoculated with *H. seropedicae* and cultivated in 0.3 mM or 3 mM of N were compared to FPKM values obtained from mock-inoculated maize plants. Differentially expressed genes (DEGs) were considered when showing significantly (p-value < 0.05) different transcript levels between treatments, using tuxedo protocol for maize and DESeq2 R package for bacteria.

### 2.6 Functional assignment and metabolic pathway analysis

To assign putative functions to DEGs in this study, the functional annotation of maize genome (*Zea mays* B73 AGPv4) was retrieved from Phytozome v. 12.1.6 (Goodstein et al., 2012) and Maize Genomics Resource (Hoopes et al., 2019), and the functional annotation of *H. seropedicae* was retrieved from KEGG and UNIPROT database. The DEGs of maize and *H. seropedicae* were annotated in the KEGG and GO database. Genes encoding proteins involved in hormone biosynthesis, receptor, signaling, and response were identified using the Arabidopsis Hormone Database v.2 (Jiang et al., 2011). Genes involved in cell wall pathways were identified using the cell wall genomics (https://cellwall.genomics.purdue.edu/) and CAZy (http://www.cazy.org/) databases. Genes encoding proteins involved in N transporter and metabolism were identified using the Arabidopsis homologs present in the list of (Wang et al., 2019) and also those that were annotated in KEGG. For the enrichment analysis of functional categories, DEGs were classified according to Gene Ontology (GO) terms and KEGG pathway using OmicShare tools (http://www.omicshare.com/tools). A GO term or KEGG pathway was considered significantly enriched if the p-value < 0.05.

### 2.7 Real-time quantitative PCR

Total RNA samples were treated with DNAse I (Biolabs), including both those utilized for RNA-seq (two replicates) and an additional third replicate intended for validating the gene expression observed in the RNA-seq. Additionally, these samples encompass those from the complementary experiment conducted. Reverse transcription was conducted, according to the manufacturer’s instructions, using superscript reverse transcriptase III (Invitrogen) with random hexamers as primer. The expression of maize individual genes was measured with gene-specific primers by real time PCR analysis with a 7500 Real-Time PCR System (Thermo Fisher Scientific) and SYBR Green PCR Master Mix (Thermo Fisher Scientific). For plant samples the relative expression was quantitated with the 2^−ΔΔCt^ calculation as previously described (Livak and Schmittgen, 2001) by using the average of expression from *28S rRNA*. The quantification of bacterial cells inside maize plants was also evaluated by molecular approach. Primer pairs designed specifically to detect *23S rRNA* genes of *H. seropedicae* ZAE94 were used (**Supplementary Table S3**). Total RNA from inoculated and mock-inoculated plants was used to compare the efficiency of inoculation. All primers used in the quantitative reverse transcription-polymerase chain reaction (qRT-PCR) are described in **Supplementary Table S3**.

## 3 Results

### 3.1 Interaction between maize and *H. seropedicae* strain ZAE94, cultivated under different N concentrations

To understand the plant-bacteria-environment relationship, we used the maize-*Herbaspirillum*-different doses of nitrogen as a model. First, a hydroponic model was established to investigate the interaction between maize and *H. seropedicae*, cultivated under different N concentrations of 0.3 mM and 3 mM (**Figure 1A**). Initially, pre-germinated maize plantlets were inoculated with the *H. seropedicae* strain ZAE94. Subsequently, these plantlets were transferred to hydroponic cultivations with either i) 0.3 mM of N or ii) 3 mM of N (**Figure 1A**).

To validate the association between maize and PGPB under our experimental conditions, root colonization by *H. seropedicae* ZAE94 was assessed 18 DAI. This was achieved by quantifying the relative expression of ribosomal RNA 23S using qRT-PCR (**Figure 1B** and **Supplementary Table S3**). Mock plants in both treatments (0.3 mM or 3 mM of N) showed low amounts of expression, indicating that maize seeds were inherently colonized by endophytic bacteria. Thus, the source of bacterial transcripts in the CTN treatments, not inoculated with *Herbaspirillum*, comes from the natural colonization of maize seeds by *H. seropedicae*. However, relative expression of *H. seropedicae* 23S was higher in inoculated plants, indicating that greater numbers of this bacteria colonized maize roots 18 DAI, compared to mock control plants, at both N concentrations (**Figure 1B**). These results confirmed that maize plants were associated with *H. seropedicae* after 18 DAI.

Since beneficial plant associations with PGPB are known to enhance leaf chlorophyll and carotenoid content (Hamid et al., 2021), these parameters were quantified in maize plants at 18 DAI (**Figures 1C, D**). There was no significant difference in chlorophyll and carotenoid contents observed between inoculated and mock plants for both N doses. However, there was a strong tendency for inoculated plants to have higher contents of photosynthetic pigments compared to non-inoculated controls (**Figures 1C, D**).

### 3.2 RNA-seq profiling of roots of maize seedlings interacting with *H. seropedicae* in two different doses of nitrogen

To investigate how maize and associated *H. seropedicae* respond to cultivation of different doses of N, we next assessed the co-expression responses of maize seedlings and *H. seropedicae* in the presence of N. Gene expression profiles of both organisms were analyzed using RNA-seq with ribosomal RNA depletion methodology. mRNA libraries of maize roots were generated and sequenced using Illumina RNA-seq. In total, eight RNA-seq libraries were sequenced: maize tissues inoculated with *H. seropedicae* (IN) and grown in either 0.3 mM (N+) or 3 mM (N-) of N, as well as mock-inoculated plants (CT) grown under the same N concentrations. The transcript abundance of maize genes was quantified through Cufflinks and calculated as FPKM. A total of 35.198 maize genes were expressed in our samples out of 41.942 genes annotated in the iGenome database. For the transcript abundance of *H. seropedicae* genes, it was used bedtools and all genes (4.840) annotated in the NCBI were expressed in our samples.

To identify differentially expressed genes (DEGs) in maize, three comparisons were analyzed: **(1)** CTN+ vs CTN-, revealing a total of 1061 DEGs (285 down-regulated and 776 up-regulated); **(2)** INN+ vs CTN+, which showed 1040 DEGs (749 down-regulated and 291 up-regulated); and **(3)** INN- vs CTN-, resulting in a total of 498 DEGs (293 down-regulated and 205 up-regulated) (**Figure 1E** and **Supplementary Table S4**). Notably, when inoculated plants were grown in the presence of a higher N concentration, a greater number of genes exhibited differential regulation compared to those grown in a lower N concentration. In the INN+ vs CTN+ comparison, 72% of genes were down-regulated, in contrast to 58.8% in INN- vs CTN- comparison. These results suggest that the inoculation with higher doses of N can lead to a down-regulation of a greater number of genes, when compared to inoculation at lower doses. In contrast, maize plants grown at higher doses of N and without inoculation showed 73% of DEGs being up-regulated. Among the three comparisons, only 18 DEGs were found to be common, and the majority of these genes exhibited the same expression pattern in both CTN+ vs CTN- and INN- vs CTN- comparisons, while the INN+ vs CTN+ comparison showed DEGs with contrasting expression patterns (**Figure 1E**). It is noteworthy that the same DEGs identified in both CTN+ vs CTN- and INN- vs CTN- comparisons share the same expression pattern, suggesting that *H. seropedicae* inoculation in lower doses of N could share some similar responses as N fertilization in maize. Conversely, the equal DEGs in CTN+ vs CTN- and INN+ vs CTN+ comparisons demonstrate contrasting expression patterns, suggesting that plants would be less responsive to *H. seropedicae* inoculation in higher doses of N, and the predominant expression profile reflected plant responses to higher N fertilization (**Figure 1E**).

To analyze the global changes in *H. seropedicae* ZAE94 generated in response to two different doses of N, two comparisons were analyzed: (1) INN+ vs CTN+: a total of 482 (382 down-regulated and 100 up-regulated) DEGs were found; and (2) INN- vs CTN-: a total of 111 DEGs (38 down-regulated and 73 up-regulated) were found (**Figure 1F**). Notably, *H. seropedicae* ZAE94 associated with maize plants grown in the presence of a higher N concentration, a greater number of genes exhibited differential regulation compared to those grown in a lower N concentration. In the INN+ vs CTN+ comparison, 79.2% of genes were down-regulated, in contrast to 34.2% in INN- vs CTN- comparison. These results suggest that higher doses of N can lead to a down-regulation of a greater number of *H. seropedicae* ZAE94 genes associated with maize plants, when compared to lower doses. Additionally, the limited overlap of only 15 DEGs between the two comparisons (**Figure 1F**) suggests that *H. seropedicae* ZAE94 shows specific responses to different N doses, with many genes exhibiting contrasting expression patterns.

To better understand the influence of different N doses in the plant-bacteria interactions, from both plant and bacterial standpoints, a functional annotation of DEGs in the GO and KEGG databases was performed. Our findings revealed that within the GO database, 66.5% of the total maize DEGs and 60.4% of the *H. seropedicae* ZAE94 DEGs were categorized into functional groups. Additionally, the KEGG database revealed that 32.6% of maize DEGs and 48.8% of bacteria DEGs were mapped to specific pathways (**Figure 1G** and **Supplementary Table S4**). This analysis provides valuable information on the specific biological processes and pathways affected by both N availability and the presence of PGPB, further enhancing our understanding of the intricate interactions between maize and *H. seropedicae* ZAE94. In addition, it also brings valuable information about the genetic response of bacteria associated with maize cultivated at different doses of N.

The GO classification was employed to categorize the function of DEGs into three main categories: cellular component, molecular function and biological processes. In maize, the comparisons yielded the following results: (1) For the CTN+ vs CTN- comparison a total of 704 (66.4%) DEGs were classified into 36 functional subcategories (**Supplementary Table S5**); (2) INN+ vs CTN+ a total of 714 (68.7%) DEGs were classified into 37 functional subcategories (**Supplementary Table S5**); (3) INN- vs CTN- a total of 321 (64.5%) DEGs were classified into 36 functional subcategories (**Supplementary Table S5**). The GO analysis revealed that most DEGs from the CTN+ vs CTN- and INN+ vs CTN+ were annotated with the same GO terms. However, when the gene set from CTN+ vs CTN- is up-regulated for a specific GO term, the one from the INN+ vs CTN+ comparison is down-regulated, and vice versa. This suggests a suppression of crucial pathways in plants inoculated with diazotrophic bacteria and cultivated with higher doses of N (**Supplementary Figure S1** and **Supplementary Table S5**). For *H. seropedicae*, the comparisons yielded the following results: (1) INN+ vs CTN+: a total of 322 (66.8%) DEGs were classified into 31 functional subcategories (**Supplementary Figure S2** and **Supplementary Table S6**); (2) INN- vs CTN-: a total of 60 (54%) DEGs were classified into 23 functional subcategories (**Supplementary Figure S2** and **Supplementary Table S6**). Most genes were categorized under identical GO terms in the two comparisons analyzed. However, in nine GO terms, the majority of annotated genes exhibited an up-regulated expression pattern in one comparison, while the other comparison showed a down-regulated pattern. This suggests that higher N concentration can differently regulate important *H. seropedicae* genes and possibly interfere with the success of the plant-bacteria association.

### 3.3 Nitrogen metabolism in maize associated with *H. seropedicae* when fertilized with two different doses of nitrogen

**Figure 2:**
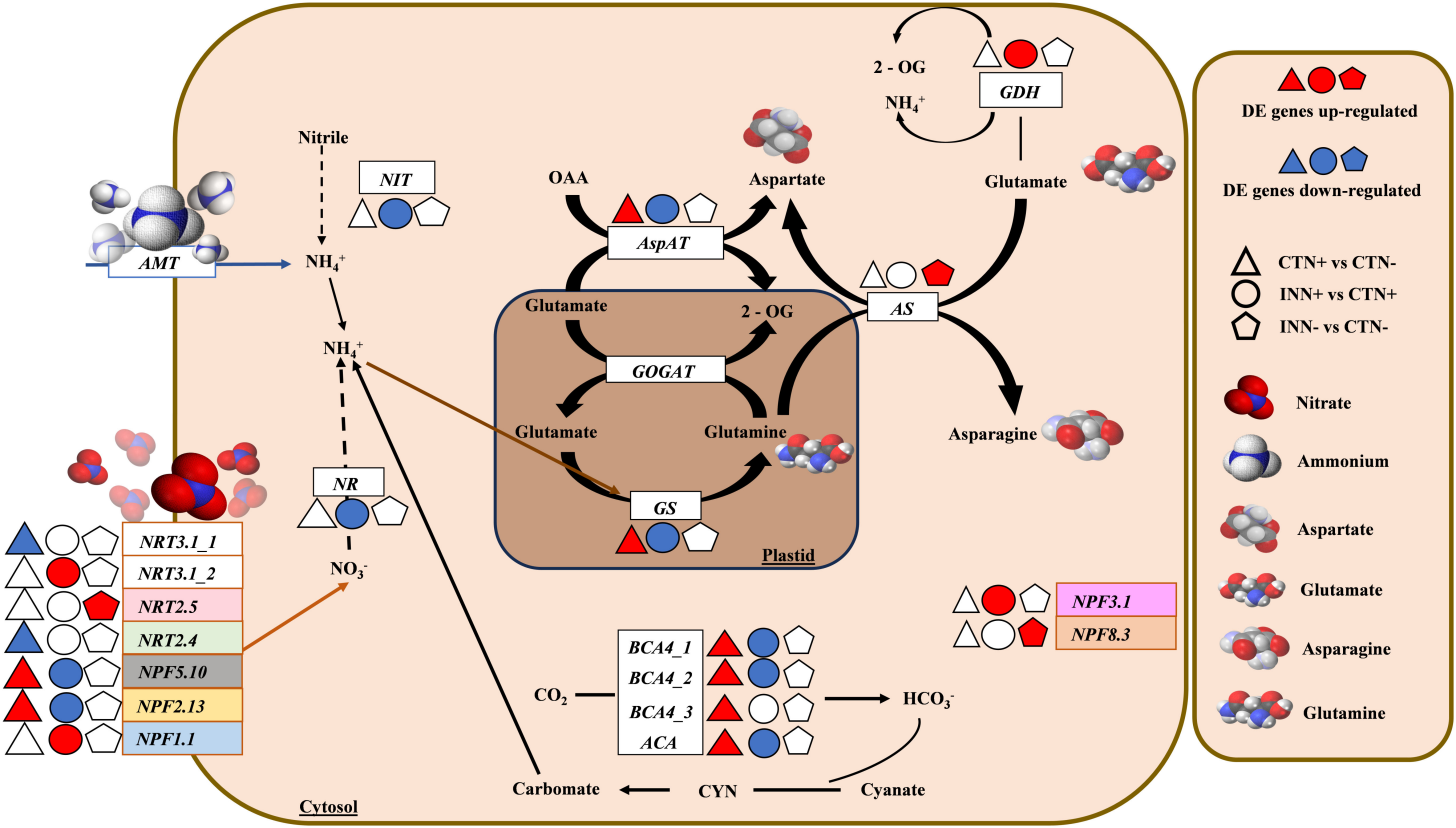
Overview of a scheme showing the regulation of nitrogen metabolism. This model presents the differentially expressed genes of maize plants inoculated with *H. seropedicae* ZAE94 (IN) or mock-inoculated (CT), and cultivated in 0.3 mM (N-) or 3 mM (N+) of nitrate. Up-regulated (red colors) or down-regulated (blue colors). White Symbol represents expressed genes, but not differentially regulated. *NIT*, nitrilase; *NRT*, nitrate transporter protein; *NPF*, nitrate transporter 1/peptide transporter family; *AMT*, ammonium transporter; *GOGAT*, glutamate synthase; *GS*, glutamine synthetase; *NR*, nitrate reductase; *BCA*, beta carbonic anhydrase; *ACA*, alpha carbonic anhydrase; *AspAT*, aspartate aminotransferase; *AS*, asparagine synthase; *GDH*, glutamate dehydrogenase; *CYN*, cyanate hydratase; *2-OG*, 2-oxoglutarate; *OAA*, oxaloacetate.

Nitrogen (N) is crucial for maize, acquired as nitrate (NO_3_^-^) or ammonium (NH_4_^+^). Our investigation highlights the significant impact of *H. seropedicae* on N metabolism in maize plants, especially in the presence of different doses of N. The expression patterns of several genes related to N assimilation, transport, and utilization were found to be differentially regulated in response to both the presence of *H. seropedicae* and varying N availability. Among the 20 DEGs identified within the N metabolism, most are associated with the *NRT/PTF* family (NPF) (**Figure 2** and **Supplementary Table S7**).

**Figure 3:**
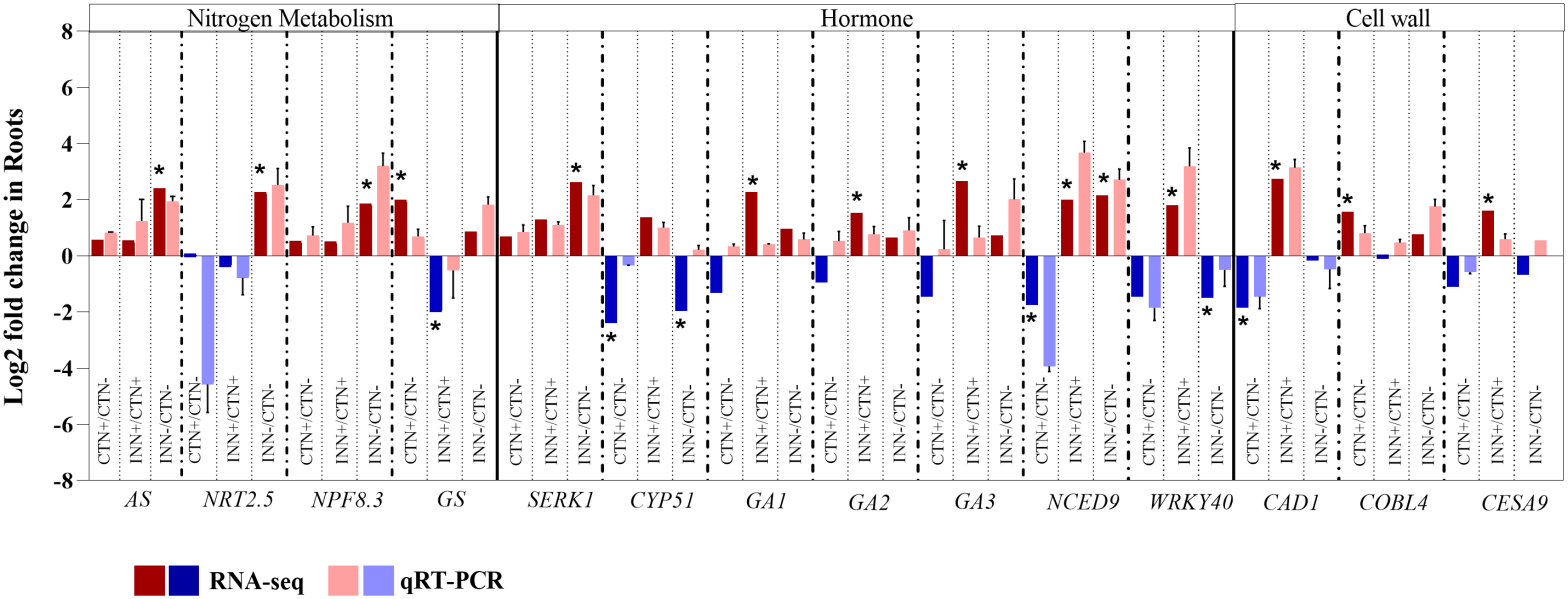
Validation of RNA-seq results by qRT-PCR analysis for maize roots. Transcript abundance pattern of selected differentially expressed genes within nitrogen metabolism, hormone synthesis and response and cell wall metabolism categories are shown for RNA-seq and qRT-PCR analyses. qRT-PCR results are presented as the ratio of expression of each gene relative to the constitutive expression of *28S rRNA* gene in each treatment when compared to mock-inoculated plants. Values are transformed into log2 fold change. Bars represent mean ± standard deviation of the relative mRNA expression in three biological replicates and each biological replicate analyzed with three technical replicates. Means with asterisk are significantly different at 5% level of confidence (Bengamini-Hochberg correction for multiple-test3).

For instance, in the comparison between non-inoculated control plants grown under different N concentrations (CTN+ vs CTN-), most of the identified DEGs were up-regulated (**Figure 2** and **Supplementary Table S7**). Notably, genes encoding high-affinity transporters, *NRT3.1* and *NRT2.4*, were down-regulated in the presence of higher N concentrations, whereas low-affinity transporters, *NPF2.13* and *NPF5.10*, were up-regulated. In addition to N transporters, several other genes exhibited differential regulation and greater expression in CTN+, including glutamine synthase (*GS)* (encodes a key enzyme in N assimilation and remobilization) (Li et al., 2017), aspartate aminotransferase (*AspAT*) (a marker of N use efficiency) (Cañas et al., 2010), GATA TRANSCRIPTION FACTOR 21 (*GATA21*) (involved in chlorophyll biosynthesis modulation), glutamate synthase (*GLU1/Fd-GOGAT*) (Hudson et al., 2011), and alpha and beta carbonic anhydrases (*ACA/BCA)*. The increased expression of genes like *NRT2.13*, *NPF5.10*, *GS*, *AspAT* and *GATA21* suggests enhanced N utilization and chlorophyll biosynthesis in plants cultivated with higher N availability. To confirm the overall expression pattern observed by RNA-seq data, the *GS* gene was selected and validated by qRT-PCR (**Figure 3**).

A higher number of DEGs involved in N metabolism were found in inoculated plants growing in higher N (INN+ vs CTN+) and most of them were down-regulated (**Figure 2** and **Supplementary Table S7**). Intriguingly, contrasting profiles were observed in the comparison of INN+ vs CTN+ compared to CTN+ vs CTN-, in which DEGs such as *NPF5.10*, *NPF2.13*, *GS* and *AspAT* exhibited a negative regulation in INN+ vs CTN+ and positive regulation in CTN+ vs CTN-. These results indicate that the presence of *H. seropedicae* induces unique responses compared to uninoculated plants, especially when higher N doses are present. Also, genes such as *NRT3.1*, which encodes a nitrate transporter that acts in low-N concentrations, and *NPF3.1*, which is involved in the transport of GAs in plants under conditions of low-N content (David et al., 2016), were more expressed in INN+. The *NR* gene encoding a cytosolic isoform of nitrate reductase, which is involved in the first step of N assimilation, was less expressed in INN+. Carbonic anhydrase genes, *ACA* and *BCA*, known for their role in legume roots with a potential role in biological nitrogen fixation (BNF) (Wang et al., 2021), were also less expressed in INN+. Nevertheless, their function in grasses with BNF remains relatively unexplored. Together, these data indicate that N metabolism can be differentially regulated, being less active in plants inoculated and grown in N+ compared to control plants grown in N+.

Lastly, in the comparison of inoculated plants with low-N levels (INN- vs CTN-) all DEGs were more expressed in inoculated plants (**Figure 2** and **Supplementary Table S7**). *NRT2.5*, which encodes a low-affinity nitrate transporter, has been shown to play a role in the plant responses independent of N uptake. Furthermore, it has been demonstrated that *NRT2.5* is essential in promoting plant growth by beneficial bacteria in *A. thaliana* (Kechid et al., 2013). *NPF8.3* encodes a di- and tri-peptide transporter that recognizes a variety of different amino acid combinations (Rentsch et al., 1995; Chiang et al., 2004), suggesting a possible active role in the N assimilation pathway, which generates amino acids. Additionally, *AS* encodes the enzyme asparagine synthetase, a key enzyme in the production of the amino acid asparagine, which is crucial for primary N metabolism (Hwang et al., 2011). *AS* was also induced in plants inoculated with low-N levels (INN-). These findings suggest that the presence of *H. seropedicae* can enhance N assimilation even in N-limited environments. qRT-PCR demonstrated greater expression of *AS*, *NRT2.5* and *NPF8.3* (**Figure 3**), validating the expression pattern described in the RNA-seq analysis.

### 3.4 Modulation of plant hormonal signaling by nitrogen fertilization and association with *H. seropedicae*

**Figure 4:**
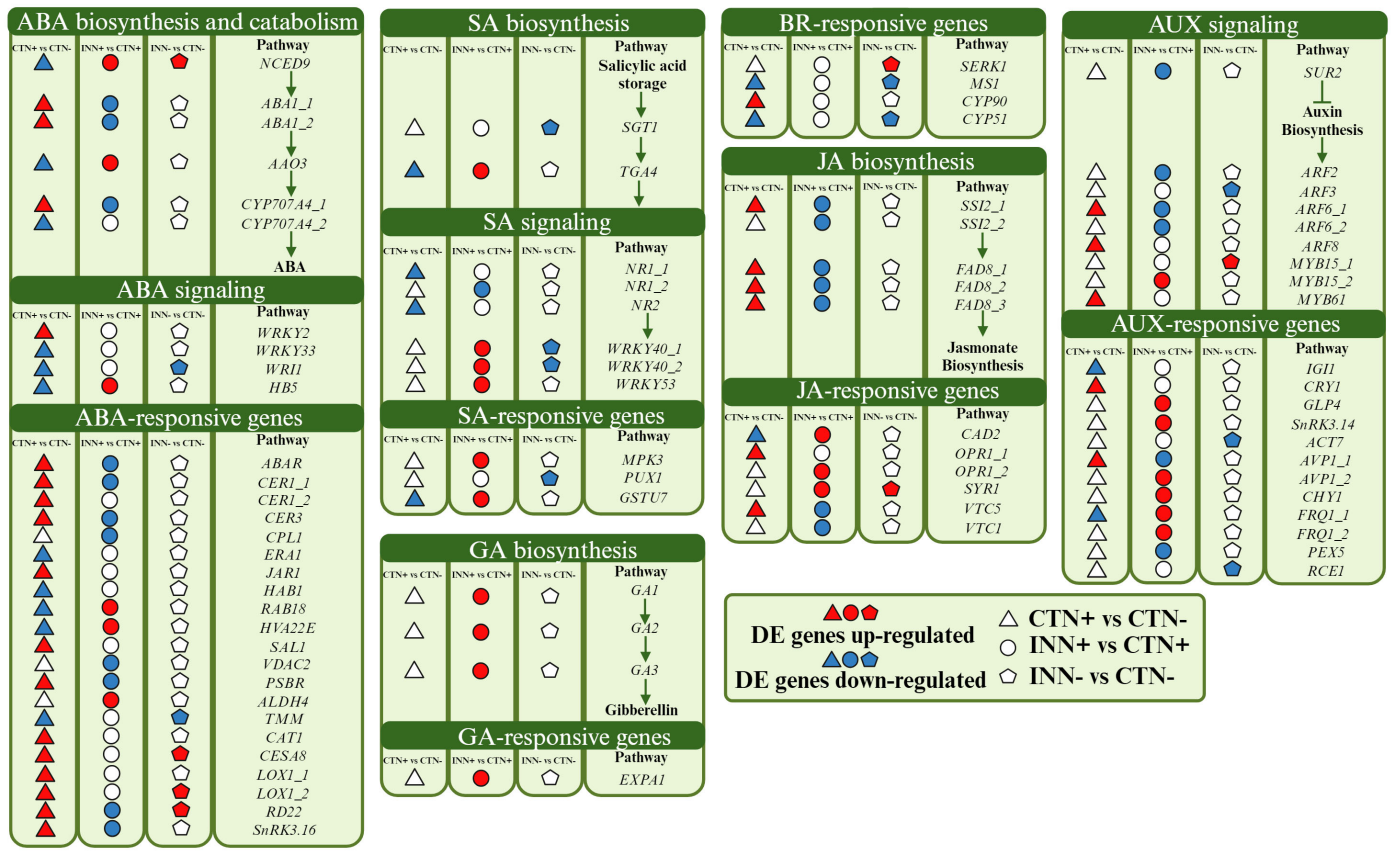
Overview of a scheme showing the regulation of hormone pathways. This model presents the differentially expressed genes of maize plants inoculated with *H. seropedicae* ZAE94 (IN) or mock-inoculated (CT) and cultivated in 0.3 mM (N-) or 3 mM (N+) of N. Up-regulated (red colors) or down-regulated (blue colors). White Symbol represents expressed genes, but not differentially regulated. ABA, abscisic acid; GA, gibberellin; AUX, auxin; JA, jasmonic acid; SA, salicylic acid; BR, brassinosteroid. Created with bioRender.com accessed on 01 December 2023.

In addition to promoting plant-growth, some PGPB can produce essential phytohormones for plant development, as well as modulate endogenous plant hormonal signaling. In order to understand whether the difference in N availability and the presence of *H. seropedicae* regulate maize genes involved in phytohormone signaling, we categorized DEGs related to different phytohormones pathways. In the three comparisons studied, 115 DEGs related to eight groups of plant hormones auxin (AUX), gibberellic acid (GA), brassinosteroid (BR), abscisic acid (ABA), jasmonic acid (JA) and salicylic acid (SA) were found (**Figure 4** and **Supplementary Table S8**).

Auxins and gibberellins, plant growth hormones produced by *H. seropedicae* (Irineu et al., 2022), influence the regulation of plant genes involved in various pathways. Remarkably, these two hormonal pathways showed higher numbers of DEGs in the comparison INN+ vs CTN+. Auxin is a phytohormone regulating key plant developmental processes. The auxin-regulating gene SUPERROOT2 (*SUR2*), encoding a Cytochrome P450 monooxygenase (CYP83B1), was repressed in INN+ vs CTN+. In Arabidopsis, the mutant of *SUR2* showed a block in the synthesis of glucosinolates leading to accumulation of the metabolic intermediate indole-3-acetaloxime (IAOx), which can be converted into indole-3-acetic acid (IAA), being an alternative route for auxin synthesis (Zhao, 2014). This data suggests that the repression of *SUR2* could contribute to activating the auxin biosynthesis pathway in maize roots inoculated with bacteria, cultivated under 3 mM of N. On the other hand, some members of the auxin response factors (ARFs) (*ARF2*, *ARF3* and *ARF6*), a family of transcription factors that regulate expression of early auxin responsive genes, were repressed in inoculated plants. Accordingly, a global activation of the auxin-responsive genes was identified in the transcriptome for the INN+ vs CTN+ suggesting that, under high N conditions, the bacteria may be promoting maize growth. It is worth highlighting the positive regulation of Arabidopsis Vacuolar H^+^-Pyrophosphatase 1 (*AVP1*), which encodes a vacuolar H^+^-pyrophosphatase that pumps H^+^ into the vacuole. Gibberellin (GA) is another phytohormone promoting plant growth (Zou et al., 2019; Qin et al., 2022). All DEGs related to the gibberellin biosynthesis pathway were up-regulated in INN+ vs CTN+ comparison, including *GA1*, *GA2* and *GA3*. These three genes are involved in the initial step of gibberellin synthesis: *GA1* encodes ent-copalyl diphosphate synthase (CPS), *GA2* encodes ent-kaurene synthase (KS) and *GA3* encodes ent-kaurene oxidase (KO) (Sun, 2008), suggesting that the levels of this hormone were elevated in plants inoculated in higher levels of N (3 mM). The RNA-seq expression profiles for *GA1*, *GA2* and *GA3* were confirmed by qRT-PCR (**Figure 3**). The GA-responsive gene, Expansin 1 (*EXPA1)*, was also up-regulated in INN+ vs CTN+. Expansin proteins are known for their multifaceted roles in plant growth, especially cell walls. Overexpression of *EXPA1* was accompanied by changes in the chemical composition of the cell wall leading to changes in root development in Arabidopsis seedlings (Samalova et al., 2023), suggesting that *EXPA*, together with several genes encoding enzymes involved in cell wall remodeling, may be part of the regulatory network involved in elongation/cell expansion, regulated by GA.

Brassinosteroids participate in the regulation of various developmental processes, including root and shoot growth, vascular differentiation, and seed germination, as well as in responding to environmental stresses (Bajguz et al., 2020). Notably, DEGs in the BR pathway were not observed in the INN+ vs CTN+ comparison. Although the regulation of a specific BR pathway was not observed, SOMATIC EMBRYOGENESIS RECEPTOR KINASE 1 (*SERK1)*, a BR-responsive gene, was induced in INN- vs CTN- and the RNA-seq expression profile for *SERK1* was confirmed by qRT-PCR (**Figure 3**). *SERK* has been proposed to mediate the interaction between BR and PAMP signaling, with evidence that the growth inhibitory effect of PAMP perception does not operate through BR signaling antagonism (Albrecht et al., 2012). Thus, *SERK1* could be also associated with the regulation of root and shoot growth, acting as a positive regulator. In the INN- vs CTN- comparison it was observed a down-regulation of a cytochrome P450, the *CYP51*, which catalyzes a step of BR-dependent sterol biosynthesis, acting decisively in the activation of defense against pathogens (Nelson and Werck-Reichhart, 2011).

ABA, recognized as a growth inhibitor, accumulates under a variety of stress conditions (Brookbank et al., 2021). Interestingly, ABA-related DEGs were predominantly found in the CTN+ vs CTN- comparison. 9-cis-epoxycarotenoid dioxygenase (*NCED*), a DEG in all comparisons, catalyzes xantoxin production, the first regulatory step in ABA biosynthesis. The xantoxin is converted to abscisic aldehyde, which is oxidized to ABA (Seo and Koshiba, 2002). *NCED* was repressed in the control situation, and it was induced in the presence of the bacteria, both in 3 mM and in 0.3 mM of N supplementation, suggesting a possible increase in ABA levels in response to inoculation. The RNA-seq expression profiles for *NCED* were confirmed by qRT-PCR (**Figure 3**). In addition, Abscisic Aldehyde Oxidase 3 (*AAO3*) is a key enzyme in the ABA biosynthesis pathway, described as up-regulated after treatment with phytohormones (ABA, auxin and GA), abiotic stress (such as saline, osmotic and cold) and biotic stress in Arabidopsis (Chan, 2012). Here, we observed a down-regulation of this gene in the CTN+ vs CTN- comparison, however it was induced in INN+ vs CTN+, which may suggest an increase in ABA biosynthesis in plants inoculated in higher levels of N (3 mM). The negative regulation of the ABA biosynthesis pathway verified in the CTN+ vs CTN- comparison reinforces the need for N availability to ensure healthy plant growth. Repression of the transcription factor *HB5* expression, a positive regulator of the ABA signaling pathway, in this comparison also suggests a decrease in the inhibitory effect of ABA on growth during seedling development. On the other hand, the positive regulation of this pathway in INN+ vs CTN+ could indicate that the presence of the bacteria constitutes a stress for the plant by increasing ABA levels. However, Cohen et al. (2015) reported that, during association with maize, PGPB such as *Azospirillum* spp., can promote plant growth through several mechanisms, including stimulating the production of phytohormones such as ABA, IAA and GA, not necessarily inducing a defense response.

JA is another hormone related to plant development and plant response to stresses (Bari and Jones, 2009). Genes associated with the JA biosynthesis pathway, *FAD8* and *SSI2*, were induced only in the CTN+ vs CTN- comparison, and showed a repressed expression pattern comparing INN+ vs CTN+. Fatty acid desaturases (FADs) introduce a double bond in the acyl chain of fatty acids, resulting in unsaturated fatty acids participating in the first steps of JA synthesis and playing essential roles in response to biotic and abiotic stresses (Hajiahmadi et al., 2020). Previous work in Arabidopsis showed that *fad3–2 fad7–2 fad8* plants showed reduced defense response to root rot caused by the root pathogen *Pythium mastophorum*, while wild-type plants were not affected by this fungus (Reymond and Farmer, 1998). As a result of this regulation, we verified the down-regulation of JA-responsive genes in INN+ vs CTN+, such as *VITAMIN C DEFECTIVE 1 (VTC1)* and *VTC5*. These genes are important to maintain the cellular reduction/oxidation (redox) homeostasis by detoxifying the cellular excessive reactive oxygen species (ROS) beyond modulates the plant development and cell growth (Song et al., 2019). In our results, the JA biosynthesis pathway was entirely repressed in INN+ vs CTN+ and not regulated in INN- vs CTN-.

The other important phytohormone that shows DEGs is the SA. SA signaling is differentially regulated by members of the WRKY family of transcription factors (Eulgem and Somssich, 2007). *WRKY40* is a pathogen-induced transcription factor that was repressed in INN- vs CTN- and induced in INN+ vs CTN+. *WRKY40* had the expression profile confirmed by qRT-PCR (**Figure 3**). Overall, these results underscore the diverse hormonal responses triggered by maize when associating with PGPB under different N concentrations, emphasizing the potential activation of the defense system, especially in higher N conditions.

### 3.5 Modulation of plant cell wall metabolism by nitrogen fertilization and association with *H. seropedicae*

The cell wall plays a fundamental role in the plant perception of bacterial and other microorganisms, in addition to its involvement in growth and development (Cosgrove, 2005; Engelsdorf et al., 2018). The plant cell wall structure consists of different molecules and components, such as proteins and polysaccharides, cellulose, hemicelluloses, pectins and lignin (Höfte and Voxeur, 2017), that can be modulated during plant-bacteria interaction (Ballesteros et al., 2021). In this study we analyzed DEGs related to pathways of cell wall synthesis and assembly in maize plants inoculated with the bacteria *H. seropedicae* ZAE94, and growing in two different doses of N. A total of 124 DEGs were annotated in the different maize gene families involved in cell wall formation, differentiation and signaling (**Supplementary Table S9**).

**Figure 5:**
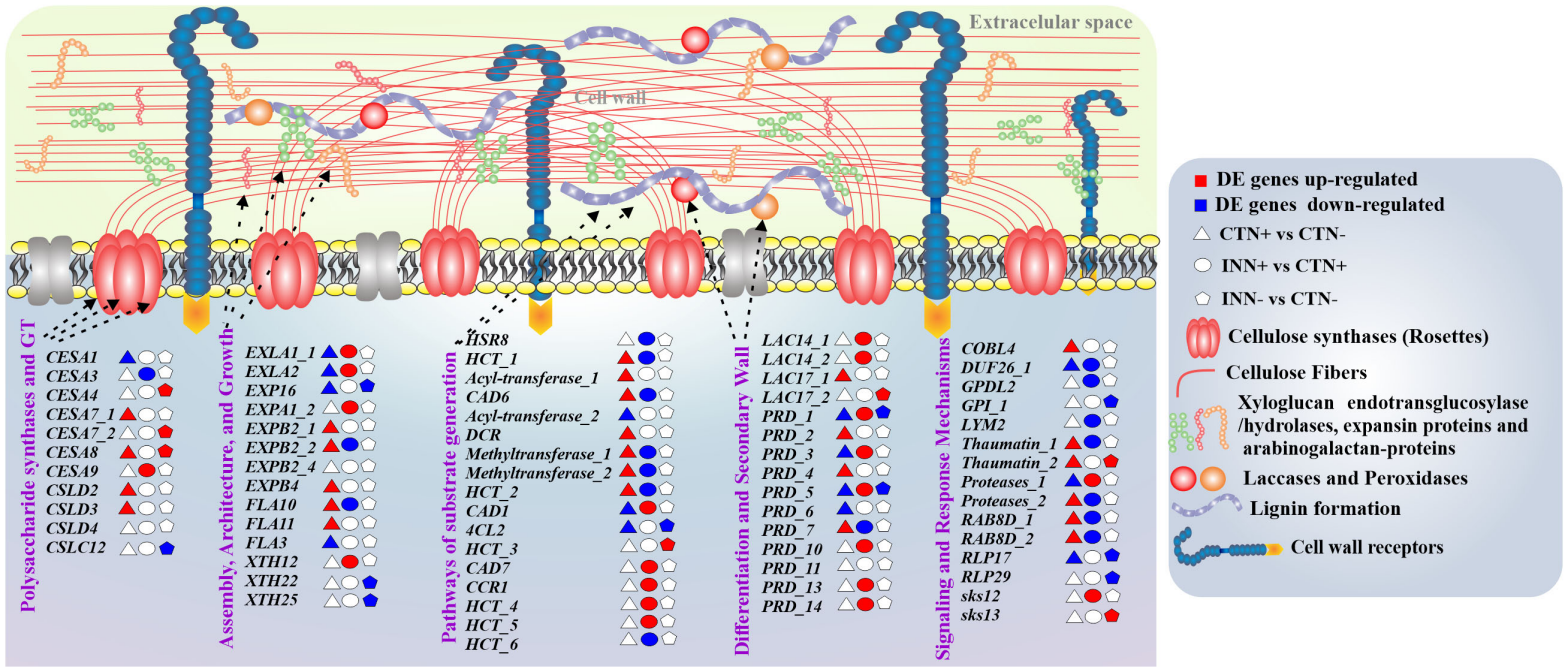
Overview of a scheme showing the regulation of cell wall components. This model presents the differentially expressed genes of maize plants inoculated with *H. seropedicae* ZAE94 (IN) or mock-inoculated (CT) and cultivated in 0.3 mM (N-) or 3 mM (N+) of N. Up-regulated (red colors) or down-regulated (blue colors). White Symbol represents expressed genes, but not differentially regulated. *CESA*, cellulose synthase, *EXLA*, expansin-like A; *EXP*, expansin; *EXPA*, expansin A; *EXPB*, expansin B; *FLA*, fasciclin-like arabinogalactan; *XTH*, xyloglucan endo-transglycosylase; *HSR8*, NAD(P)-binding Rossmann-fold superfamily protein; *HCT*, hydroxycinnamoyl-CoA shikimate/quinate hydroxycinnamoyl transferase; *CAD*, cinnamyl alcohol dehydrogenase; *DCR*, HXXXD-type acyl-transferase family protein; 4CL2, 4-coumarate-CoA ligase 2; *CCR1*, cinnamoyl-CoA reductase 1; *LAC*, laccase; *PRD*, peroxidase; *COBL4*, COBRA-like extracellular glycosyl-phosphatidyl inositol-anchored protein family; *DUF26*, Domain of unknown function; *GPDL2*, PLC-like phosphodiesterase family protein; *GPI*, Carbohydrate-binding X8 domain superfamily protein; *LYM2*, Lysm domain GPI-anchored protein 2 precursor; *RAB8D*, RAB GTPase homolog E1B; *RLP*, Leucine-rich repeat (LRR) family protein; *SKS*, SKU5 similar.

Differentially expressed genes from the Cellulose synthase (*CESA*) family showed induction and repression profiles depending on N concentration or association with bacteria (**Figure 5**). CESA proteins are divided into two subclasses, CESA1, 2, 3, 5, 6, and 9, and CESA4, 7, and 8, which are involved in the synthesis of primary and secondary cell walls, respectively (Li et al., 2020). The comparisons CTN+ vs CTN- and INN- vs CTN- showed up-regulated DEGs involved in secondary cell wall, while INN+ vs CTN+ comparison showed one DEG down-regulated (*CESA3*) and one DEG up-regulated (*CESA9*), that are involved in the synthesis of primary cell wall (**Figure 5**). These results suggest that a beneficial maize – diazotrophic bacteria association, in soils with lower levels of N, could favor the synthesis of cell wall components such as cellulose. The increase in cellulose accumulation could thicken the cell wall, making it more resistant in response to possible stressful situations such as pathogen attack and drought.

The cell wall architecture and growth pathway, important for the extension of the cell wall, showed several DEGs in the CTN+ vs CTN- comparison (**Figure 5**). The N doses can positively regulate wall extensibility since some genes, such as Expansin B2 (*EXPB2_1*), (*EXPB2_2*) Expansin B4 (*EXPB4*), FASCICLIN-like Arabinogalactan-protein 10 (*FLA10*) and FASCICLIN-like arabinogalactan-protein 11 (*FLA11*), were induced. On the other hand, in inoculated plants, the expression pattern of some genes involved in this pathway were repressed in N+ condition, such as the ones that were positively regulated in the control condition, as well as in low-N condition as Xiloglucan endotransglycosylase 22 and 25 (*XTH22* and *XTH25*) (**Figure 5**). This result suggests that bacterial association could modulate cell wall composition, leading to less enzymatic activity in some processes involved in extending and loosening the wall.

The substrate generation pathway showed several DEGs up-regulated or down-regulated in the CTN+ vs CTN- and INN+ vs CTN+ comparisons, showing contrasting expression profiles between the two comparisons. On the other hand, some genes such as cinnamyl-alcohol dehydrogenase 1 (*CAD1*) (**Figure 3**), cinnamyl-alcohol dehydrogenase 7 (*CAD7*), cinnamoyl coa reductase 1 (*CCR1*) and hydroxycinnamoyl-CoA shikimate/quinate hydroxycinnamoyl transferase (*HCT*) were induced only on INN+ vs CTN+ comparison (**Figure 5**). The induction of this group of genes may indicate greater lignification in root cells that have a secondary wall, since they are related to the synthesis and production of lignin. This lignification profile can be confirmed with the expression profile of laccase and peroxidase enzymes that are part of the secondary cell wall differentiation and synthesis pathway (**Figure 5**). Several DEGs were induced in the INN+ vs CTN+ comparison, suggesting that a high concentration of N applied to plants that were inoculated with PGPB, has a greater expression of enzymes that act in oxidation and formation of lignified compounds. On the other hand, in plants inoculated and treated with low-N, few laccases and peroxidase DEGs were regulated, and in most of them, the expression was reduced (**Figure 5**). This result may suggest that INN- vs CTN- plants could have more permeable and less rigid walls, but with more cellulose content, as previously discussed based on the expression profile of cellulose synthase genes (**Figures 3** and **5**).

In the signaling and response pathways related to cell wall, several DEGs were induced in CTN+ vs CTN- comparison, including Cobra like 4 gene (*COBL4*) Domain of unknown function (*DUF26*), *Thaumatin 1* and *2* (**Figure 3**), suggesting that plants in high N concentrations may have a more active immune system, considering that these genes are related to plant protection against biotic and abiotic stresses. On the other hand, several repressed DEGs were observed in the INN+ vs CTN+ comparison, and only the Proteases_1 and SKU5 similar 12 (*SKS12*) genes related to homeostasis of reactive oxygen species were induced (**Figure 5**) (Van der Hoorn and Klemenčič, 2021; Chen et al., 2023). In the comparison of INN- vs CTN-, only a few genes were DEGs and most showed a repression expression profile, such as glycosylphosphatidylinositol-anchored protein 1 (*GPI_1*) and two Receptor like protein (*RLPI 17* and *29*) (**Figure 5**). Possibly the repression of these genes attenuates the immune system, making the plant’s association with PGPB more efficient in conditions where plant nutrients are scarce.

### 3.6 Reprogramming of *H. seropedicae* ZAE94 metabolic subsystems in response to N availability

**Figure 6:**
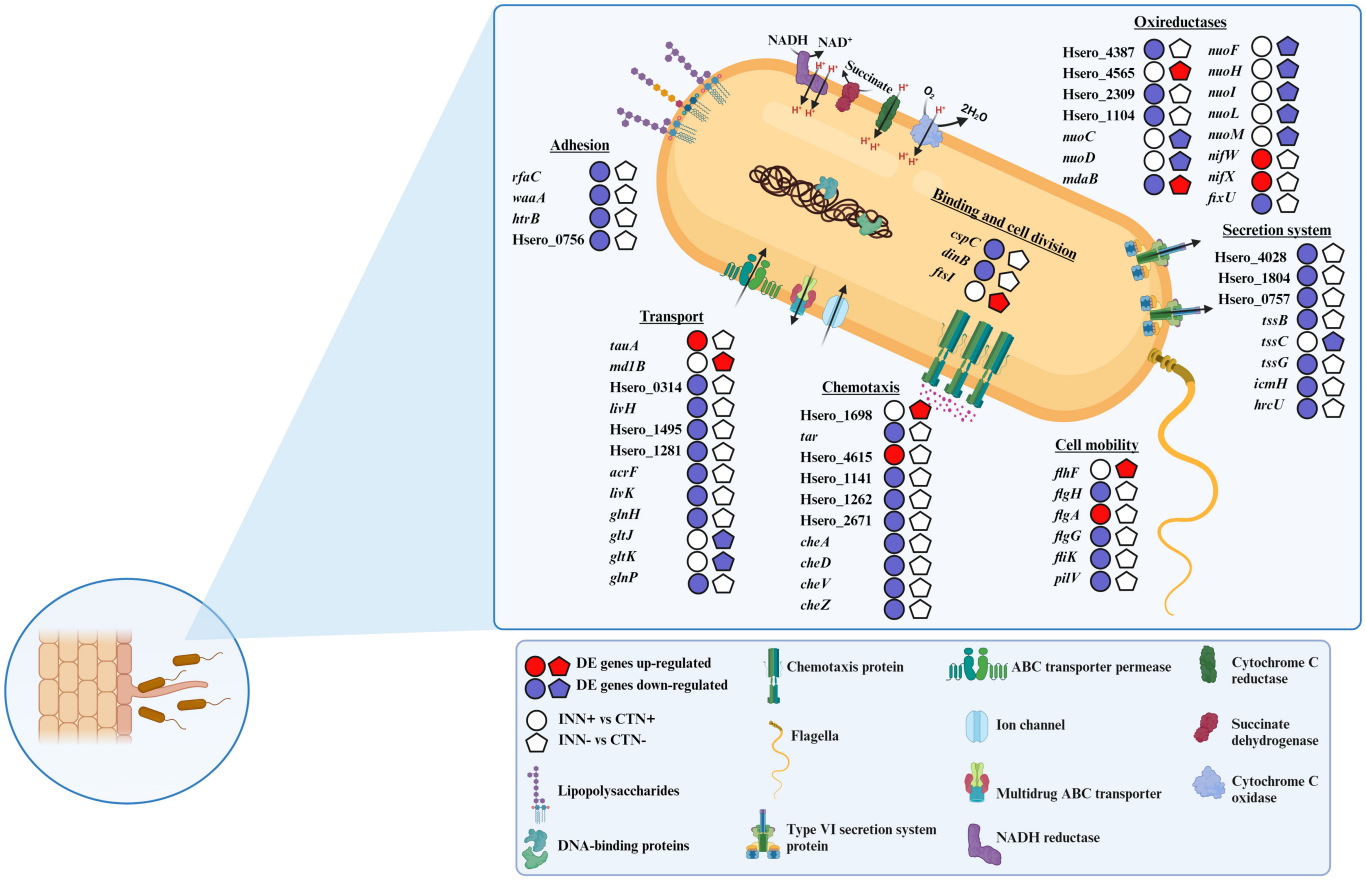
Overview of the major *H. seropedicae* strain ZAE94 metabolic pathways and functions differentially expressed in two different doses of nitrate. Pathways involved in relevant aspects of *H. seropedicae* ZAE94 as PGPB are represented, including adhesion, transport, chemotaxis, cell mobility, secretion system and oxireductases. Up-regulated (red colors) or down-regulated (blue colors). White Symbol represents expressed genes, but not differentially regulated. Created with bioRender.com accessed on 01 December 2023.

After wide identification of the functional categories of genes differentially expressed in *H. seropedicae* identified by RNA-seq, a more detailed analysis of a possible involvement of DEGs in physiological and metabolic processes of the bacteria was performed. A schematic representation of the results found is shown in **Figure 6**. In addition to checking the gene expression profile, we also analyzed the regulation of CDS, indicated based on Hsero_XXXX codes.

The association process of *H. seropedicae* with poaceous species begins with the attraction of bacteria to the host roots, subsequently the bacteria attachment on the root surfaces occurs and finally the emergence points of the lateral roots are colonized (Monteiro et al., 2012; Thiebaut et al., 2022). The RNA-seq analyses revealed that genes related to adhesion were down-regulated in the INN+ vs CTN+ comparison, including three genes coding for lipopolysaccharides (LPS) biosynthesis proteins (*rfaC*, *waaA* and *htrB*). In addition, Hsero_0756, coding for membrane porin of the OmpA family, was also repressed in INN+ treatment when compared to CTN+ (**Figure 6**). These results suggest that the adhesion process is being repressed in inoculated plants growing at higher N levels.

**Figure 7:**
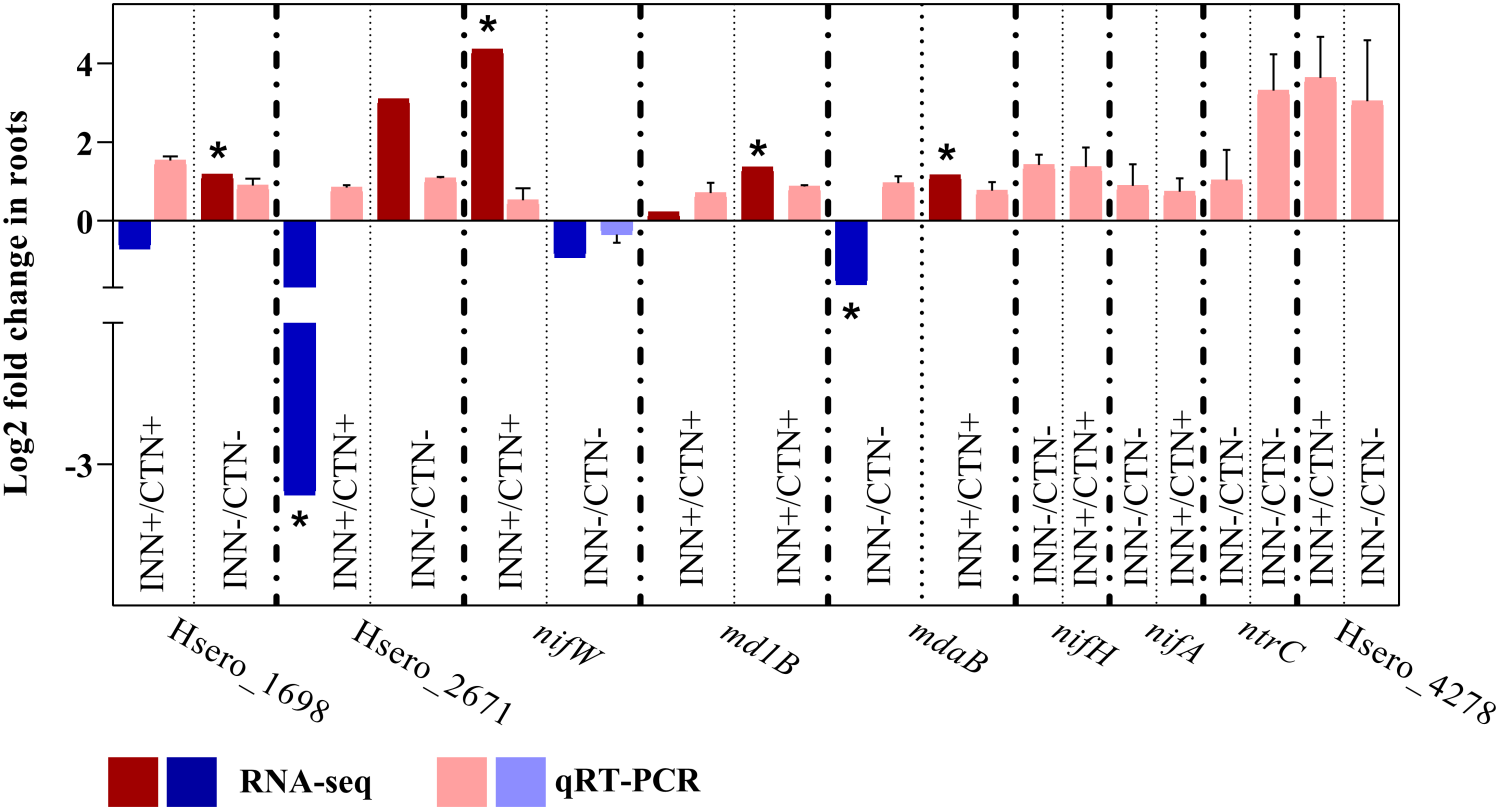
Validation of RNA-seq results by qRT-PCR analysis for *H. seropedicae* associated with maize roots and expression quantification of *nif* and *auxin* production genes in *H. seropedicae* ZAE94 colonizing maize roots by qRT-PCR. Transcript abundance patterns of selected differentially expressed genes are shown for RNA-seq and qRT-PCR analyses. qRT-PCR results are presented as the ratio of expression of each gene relative to the constitutive expression of *28S rRNA* gene in each treatment when compared to mock-inoculated plants. Bars represent mean ± standard deviation of the relative mRNA expression in three biological replicates and each biological replicate analyzed with three technical replicates. Values are transformed into log2 fold change. Means with asterisk are significantly different at 5% level of confidence (Bengamini-Hochberg correction for multiple-test3).

Regarding genes related to bacterial chemotaxis, two DEGs, Hsero_1698 and Hsero_2164, were up-regulated in INN- vs CTN- comparison. The RNA-seq expression profiles for Hsero_1698 was confirmed by qRT-PCR (**Figure 7**). The INN+ vs CTN+ comparison revealed that four *MCP* genes were significantly repressed (Hsero_4615, Hsero_1141, Hsero_1262 and Hsero_2671). The RNA-seq expression profiles for Hsero_2671 was confirmed by qRT-PCR (**Figure 7**). Moreover, several genes that encode chemotaxis-related proteins were down-regulated in this comparison: *cheA, cheD, cheV* and *cheZ* (**Figure 6**). Bacterial cell mobility has also a crucial implication in plant colonization by the plant growth promoter, since bacteria chemotactic movement in response to various stimuli and environmental changes depends on flagella-driven motility (Tadra-Sfeir et al., 2015). Regarding the INN+ vs CTN+ comparison, the flagellin gene *fliK* was significantly repressed. Moreover, two genes involved in the formation of the flagellar basal body, *flgH* and *flgG*, showed reduced expression levels (**Figure 6**). In this context, the gene *pilV*, coding for a putative type IV *pilus* assembly protein, was also repressed (**Figure 6**). However, in the condition of low availability of N, the comparison INN- vs CTN- revealed the up-regulation of *flhF* gene, coding for transcription activator protein (**Figure 6**). Taken together, the results indicate that, under low-N conditions, there is an increase in the expression of genes involved in chemotaxis and flagellar biosynthesis in *H. seropedicae* when compared to the mock group, suggesting that this condition may have positive effects on motility processes that are important for establishing association with the plant. On the other hand, a large number of genes involved in chemotaxis and motility were repressed in *H. seropedicae* under high-N conditions, suggesting that this condition is not favorable for processes important for plant-interaction.

In pathogenic bacteria, T3SS and T6SS are key secretion systems acting to transport effector proteins to host plants. Therefore, these two types of secretory systems are commonly absent or present in low abundance in beneficial endophytic bacteria (Dudeja et al., 2021). In this study, *T3SS* secretion system genes were not differentially expressed in any of the comparisons analyzed. Some *T6SS* genes were significantly down-regulated in INN+ vs CTN+ comparison: *icmH*, *hrcU, tssB, tssG*, Hsero_1804, Hsero_0757 and Hsero_4028; meanwhile only *tssC* showed a decreased expression pattern in INN- vs CTN- (**Figure 6**). Down-regulation of *tssC* and other *T6SS* genes in inoculated plants may indicate that this protein secretion system is present with reduced abundance during the colonization of maize plants by *H. seropedicae* ZAE94, reinforcing the beneficial aspect of this association for the plant.

The transcriptomic analysis revealed a large number of differently expressed transport related genes among the highlighted comparisons, including transporters of the resistance-nodulation-division (RND) family, the major facilitator superfamily (MFS) and the ATP-binding cassette (ABC) (**Supplementary Table S10**). Hsero_3390, coding for a putative nitrate ABC transporter, was induced in *H. seropedicae* ZAE94 in response to high N availability (**Figure 6**). However, several ABC transporter genes involved in amino acid transport were significantly repressed in INN+ vs CTN+ comparison, including *glnP*, *glnH*, *livK*, Hsero_4012, Hsero_1495 and Hsero_1281 (**Figure 6**). In addition, two multidrug ABC transporter genes, *acrF* and Hsero_0314, also had the expression decreased (**Figure 6**). On the other hand, just four ABC transporters were regulated in the comparison INN- vs CTN-, two glutamate/aspartate ABC transporters, *gltK* and *gltJ*, and a urea ABC transporter, *urt* were down-regulated, and the multidrug ABC transporter gene *md1B* was up-regulated (**Figure 6**). *md1B* expression levels were validated by qRT-PCR analyzes (**Figure 7**). The data shows that a large number of genes involved in transport were repressed in *H. seropedicae* under high-N conditions.

RNA-seq analyzes showed that an expressive number of genes involved in oxidoreductase activity were differentially expressed in the comparisons analyzed (**Supplementary Table S10**). *nifW* and *nifX* were significantly up-regulated, while *fixU* was down-regulated in INN+ vs CTN+ (**Figure 6**). The expression level of the *nifW* gene was also evaluated, which was up-regulated in INN+ vs CTN+ and down-regulated in INN- vs CTN- (**Figure 6**). Considering that the ability of *H. seropedicae* to promote plant growth might depend on many genes involved in N_2_ fixation (Pedrosa et al., 2011), our results suggest that, under our experimental conditions, the presence of N would be favorable to bacterial N_2_ fixation in *H. seropedicae* ZAE94. Among the DEGs with differential expression profiles in the INN+ vs CTN+ and INN- vs INN+ comparisons are 11 contra-regulated genes, including genes involved in oxidoreductase activity (**Figure 1F** and **Supplementary Table S10**). Interestingly, *mdaB* had its expression down-regulated in INN+ vs CTN+, and was up-regulated in INN- vs INN. *mdaB* expression pattern was validated in qRT-PCR analyzes (**Figure 7**). Among the genes with repressed expression in INN+ vs CTN+ comparison, there are three genes involved in the cytochrome C enzymatic chain, which is associated to bacterial microaerobic respiration: Hsero_4387, Hsero_2309 and Hsero_1104 (**Figure 6**). In particular, seven genes encoding NADH ubiquinone oxidoreductase were significantly down-regulated in INN- vs CTN- comparison, *nuoC*, *nuoD*, *nuoF*, *nuoH*, *nuoI*, *nuoL* and *nuoM* (**Figure 6**).

Among the regulated genes that have functions involved in cell division, we verified the induction of *ftsI* expression in the INN- vs CTN- comparison (**Figure 6**), which encodes an essential transpeptidase that introduces peptide cross-linking into the peptidoglycan cell wall in the division septum during cell division (Weiss et al., 1999). Meanwhile, *dinB* (Hsero_4328) had expression decreased in INN+ vs CTN+, coding a DNA polymerase (pol) IV, which is highly expressed to help bacterial cells tolerate certain types of DNA damage (Friedberg et al., 1995) (**Figure 6**). In addition, *cspC* (Hsero_0505), coding for a cold shock protein, was also down-regulated in *H. seropedicae* ZAE94 in comparison to the mock-inoculated group, under high N levels (**Figure 6**).

### 3.7 Nitrogen control of the beneficial effects of maize inoculation with *H. seropedicae*

A global view of the integrated modulation of gene expression in the maize and *H. seropedicae* association, occurring with different doses of N fertilization, was generated by the RNA-seq data. To further characterize the influence of N fertilization to the benefits to the plant, a new experiment was carried out to investigate the beneficial effects of maize inoculation with *H. seropedicae* ZAE94, cultivated in two different doses of N (0.3 mM and 3 mM).

**Figure 8:**
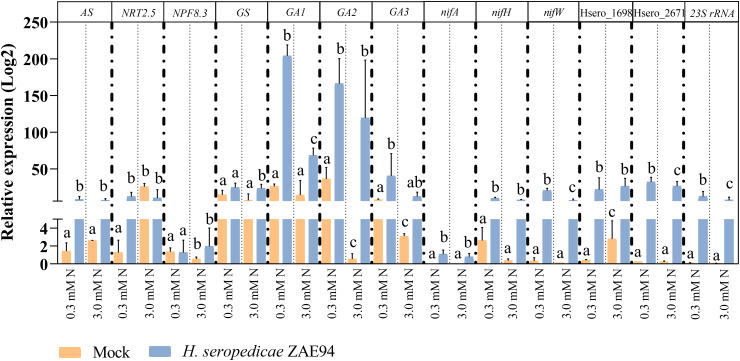
qRT-PCR analysis for genes of *H. seropedicae* associated with maize roots. Transcript abundance patterns of selected differentially expressed genes are shown for RNA-seq and qRT-PCR analyses. qRT-PCR results are presented as the ratio of expression of each gene relative to the constitutive expression of *28S rRNA* gene in each treatment when compared to mock-inoculated plants. Values are transformed into log2 fold change. Bars represent mean ± standard deviation of the relative mRNA expression in three biological replicates and each biological replicate analyzed with three technical replicates. Means with different letters are significantly different at 5% level of confidence (Two-way ANOVA test with Tukey’s post-test).

First, to confirm that maize and *H. seropedicae* were associated under the experimental conditions performed, root colonization by *H. seropedicae* ZAE94 was assessed by quantifying the relative expression of *23S rRNA* (**Figure 8**). The data validated the inoculation and suggested a better bacterial colonization in plants growing with lower dose of N.

**Figure 9:**
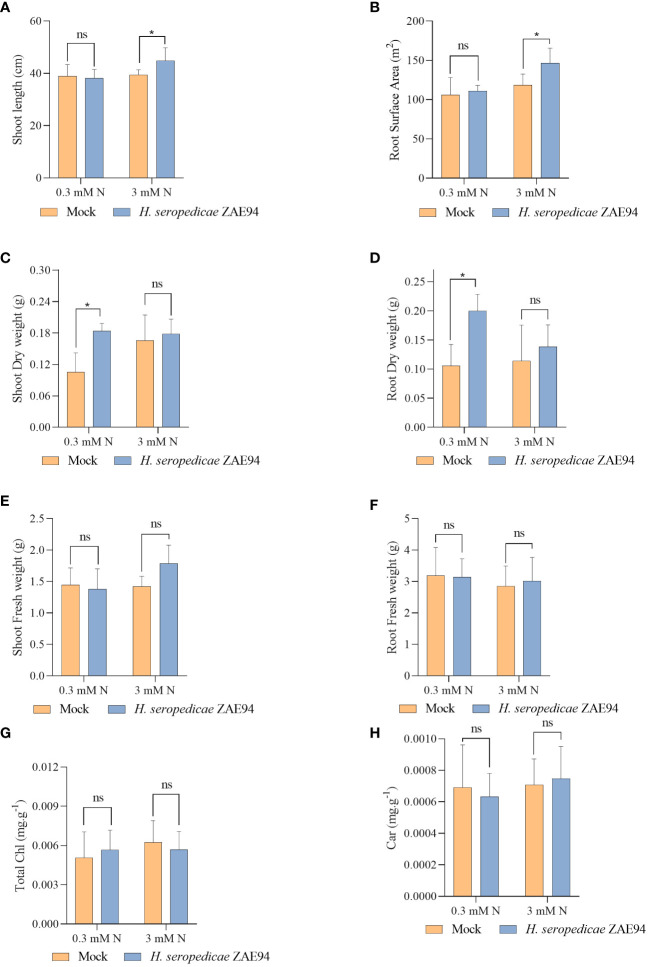
Effects of inoculation with *H. seropedicae* ZAE94 on maize plants grown in two different doses of N (0.3 mM and 3 mM of nitrate), 18 days after inoculation. **(A)** Shoot length; **(B)** Root surface area; **(C)** Shoot dry weight; **(D)** Root dry weight; **(E)** Shoot fresh weight; **(F)** Root fresh weight; **(G)** Total chlorophyll content; **(H)** Carotenoid content. Columns represent the mean value and bars represent the standard error of 10 replicates. Means with asterisk are significantly different at 5% level of confidence and means with ns were not significant (Two-way ANOVA test with Tukey’s post-test).

The effects of fertilization with two doses of N were analyzed in plants 18 DAI (**Supplementary Figure S3**). Several phenotypic parameters were evaluated to quantify the benefits of inoculation in promoting shoot and root growth, such as shoot length, fresh and dry matter, as well as the root fresh matter, in addition to the analysis of the roots surface (**Figure 9**). The parameters were measured 18 DAI, according to the harvest performed for RNA-seq analysis. Remarkably, different beneficial effects were observed in inoculated plants growing with 0.3 mM or 3 mM of N. At 18 DAI, shoots were longer in inoculated plants compared to mock plants at 3 mM of N (**Figure 9A**). Furthermore, the association with *H. seropedicae* increased total root surface area in the high-N treatment (**Figure 9B**). On the other hand, the shoot and root dry matter of inoculated plants were higher than that observed for the mock plants only in response to 0.3 mM of N (**Figures 9C, D**). Interestingly, these results suggest that the effect of inoculation in promoting biomass was greater in plants subjected to lower N concentrations, statistically equating the biomass of these plants to those observed in plants grown under higher doses of N. The inoculation had no effect on the fresh matter of the root, at all N doses evaluated (**Figures 9E, F**). Also, chlorophyll and carotenoid contents were not significantly different in response to inoculation, regardless of the N doses used (**Figures 9G, H**). Taken together, the results obtained in the new experiment validate the data obtained in RNA-seq at the level of plant development. Also, the results suggest that the reduction of doses of N fertilization commonly applied in maize crops, from 3 mM of N to 0.3 mM, allows inoculation with *H. seropedicae* to produce more significant effects in promoting plant biomass, both in aerial part and roots, probably due to an improvement in the efficiency of the plant-bacteria association.

In addition to plant growth validation, mRNA expression in genes that participate in key biological processes involved in a beneficial association for the plant were analyzed. The expression profile of four genes associated with N metabolism (*AS*, *NRT2.5*, *NPF8.3* and *GS*) was analyzed revealing higher expression levels of *NPF8.3* and *GS* in inoculated plants cultivated with 0.3 mM N (**Figure 8**). In general, the increased expression of these genes in inoculated plants, compared to control plants, suggests an improvement in N utilization, especially in plants cultivated with lower N availability. As observed in the transcriptome analyses, these results reinforce that the presence of *H. seropedicae* can promote N assimilation in N-limited environments, leading to increases in plant biomass. Furthermore, three genes involved in GA metabolism (*GA1*, *GA2* and *GA3*) were also verified, showing higher mRNA levels in inoculated plants cultivated with 3 mM N, compared to mock-inoculated seedlings, as verified in RNA-seq (**Figure 8**).

From the bacterial side, the expression patterns of five *H. seropedicae* genes, associated with chemotaxis and N_2_-fixation (*nifA*, *nifH*, *nifW*, Hsero_2671 and Hsero_1698), were evaluated in response to different doses of N, by qRT-PCR (**Figure 8**). Expression analysis of the N_2_-fixation genes, *nifA* and *nifH*, demonstrated a strong trend towards higher expression in samples cultivated under 0.3 mM N when compared to the 3.0 mM N treatment, being significantly higher in this condition for *nifW* expression (**Figure 8**). The same expression patterns were verified for the Hsero_2671 gene (**Figure 8**). These results suggest that the lowest dose of N can stimulate the bacterial metabolism, promoting the expression of *nif* genes, as well as host adhesion and recognition, in accordance to the RNA-seq data.

## 4 Discussion

Plants are remarkably sensitive organisms that respond dynamically to the environment in which they grow. Plant associations with beneficial bacteria are combined to components of the environment such as soil, water or the atmosphere, functioning as a coordinated unit to generate plant adaptive responses, improving growth (Carvalho et al., 2016). Many studies have shown that the efficiency of the plant association with PGPB is dependent on external factors, such as the plant genotype, the bacterial genotype, and the soil nutritional status (Ballesteros et al., 2021; Carvalho et al., 2022; Thiebaut et al., 2022). Among the environmental factors, the nutritional composition of the soil plays a pivotal role in shaping the growth and development of plants. The presence of essential nutrients and micronutrients profoundly impact the health and productivity of plants (Nadeem et al., 2018). Deficiencies or imbalances in these nutrients can hinder vital processes like photosynthesis, root development and overall growth (Nadeem et al., 2018). Therefore, to advance the understanding of the mechanisms involved in a beneficial association for the plant, it is important to investigate, in an integrated way, the genetic, physiological and biochemical factors in response to multiple stimuli. To address that, in this work, we studied the relationship between plant-bacteria-environment signaling, using as a model the association of maize plants with *H. seropedicae* ZAE94 and cultivated with different doses of N. Ribosomal RNA-depleted RNA sequencing was performed to generate a global view of the integrated modulation of gene expression of both maize and *H. seropedicae* ZAE94, when associated and cultivated under different doses of N. In parallel, N control of the beneficial effects of maize inoculation with *H. seropedicae* was investigated by phenotyping the development of maize inoculated plants. Taken together, our results revealed new insights into pathways involved in grass-PGPB associations, potentially offering sustainable alternatives to reduce chemical fertilizers dependence in agriculture.

**Figure 10:**
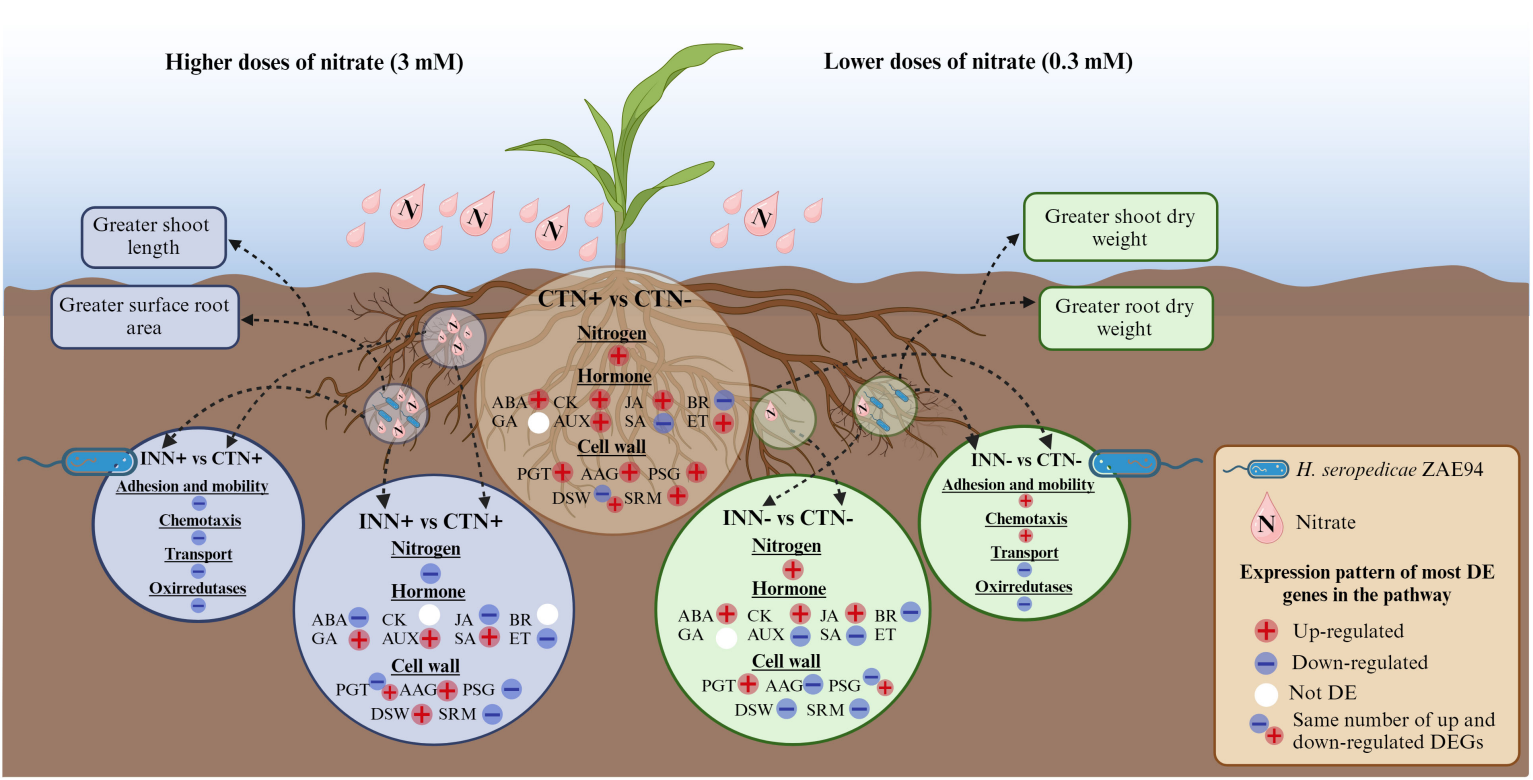
Schematic model for genetic controls modulated in response to maize - *H. seropedicae* – nitrogen interactions. This model presents a summary of the most relevant results for the differentially expressed pathways of maize plants inoculated with *H. seropedicae* ZAE94 (IN) or mock-inoculated (CT) and cultivated in 0.3 mM (N-) or 3 mM (N+) of N and promoting plant growth in each condition. See text for detailed discussion. Up-regulated (red colors) or down-regulated (blue colors). White Symbol represents that there were expressed genes in the pathway, but not differentially regulated. DE, differentially expressed; ABA, abscisic acid; GA, gibberellin; AUX, auxin; JA, jasmonic acid; SA, salicylic acid; BR, brassinosteroid; PGT, polysaccharide synthases and GT; AAG, assembly, architecture, and growth; PSG, pathways of substrate generation; DSW, differentiation and secondary wall; SRM, signaling and response mechanisms. Created with bioRender.com accessed on 01 December 2023.

To enhance our comprehension of the overall benefits arising from different doses of N, plant growth-promoting bacteria, or their combined application, we have crafted a representative figure encapsulating the comprehensive results of our study. This figure illustrates the co-regulation of specific plant and bacterial pathways when associated under different N doses (**Figure 10**).

### 4.1 The relationship between nitrogen nutritional status with maize and bacteria nitrogen metabolism

In this study, the acquisition of N seems to be induced in maize plants grown in higher doses of N or inoculated with PGPB and cultivated in low-N, since most of the genes involved in N metabolism were induced in the RNA-seq and qRT-PCR analysis. Corroborating our data, studies involving plant inoculation with PGPB have consistently shown the transcriptional induction of genes related to nitrate transporters and N metabolism enzymes. For instance, Hardoim et al. (2019) demonstrated that *A. brasilense* and *H. seropedicae* inoculation induced the expression of genes encoding nitrate transporter proteins (NRT1.11 and NRT3.1), NR, and NiR in maize. Similarly, Li et al. (2019) observed increased expression of *NRT2.1*, *NR* and *GS* in maize, along with several genes (*NRT1.1, NRT2.1, NRT2.3, NR, GS1, GOGAT*) in wheat, and others (*NRT1.3, NRT2.2, NR1, NiR, GS1, GOGAT*) in cucumber upon inoculation with *Paenibacillus beijingensis* BJ-18. *NRT* genes that were also significantly up-regulated in tomato grown in low-N and inoculated with diazotrophic *Enterobacter radicincitans* (Berger et al., 2013). Similar data was also reported in plant inoculation with beneficial fungi. Sherameti et al. (2005) showed that the inoculation of the endophytic fungus *Piriformospora indica* lead to up-regulation of *NR* gene expression, resulting in increased N accumulation in Arabidopsis and tobacco seedlings. It was also reported that the expression levels of *GS* and *NRT* genes were significantly higher in plants inoculated with the endophytic fungus *Phomopsis liquidambari* compared to uninoculated plants, under low-N (Yang et al., 2014). Notably, the enhanced expression of the genes involved in N metabolism was predominantly observed in plants inoculated and cultivated under low-N conditions rather than in those grown under high-N conditions, in accordance with the data obtained in our work.

Remarkably, functional analysis of homologs to the DEGs found in our studies, as well as other genes involved in N metabolism, lead to benefits to the plant similar to the ones provided by the PGPB. Studies on the overexpression of *AS* showed its positive impact on N uptake and assimilation, improving tolerance to N deficiency in rice (Lee et al., 2020) and greater tolerance to N limitation, accompanied by an increase in total free amino acids in *A. thaliana* (Lam et al., 2003). Ravazzolo et al. (2020) reported increased expression of genes like *GS* and *BCA4* in maize roots when grown in the presence of N, contrasting with those grown without a N source. Transgenic maize constitutively overexpressing *GLN1-3*, which encodes a cytosolic GS, showed a 30% increase in grain number (Martin et al., 2006), while GS-deficient maize mutants showed a reduction in grain yield (Cañas et al., 2010). Additionally, overexpressing *DvGS1* improved growth and N utilization in *A. thaliana* (Zhu et al., 2014). Furthermore, overexpression of *AspAT* genes in rice led to altered N metabolism and increased amino acid and protein content in seeds. Our results, combined with data from the literature, demonstrate that the positive regulation of plant N metabolism genes can contribute to plant growth benefits. Given that inoculation of PGPB under low-N conditions positively regulated the expression of crucial genes in the N pathway, it can be inferred that reducing N doses for cultivation may lead to environmentally sustainable and economically viable agriculture.

Previous studies confirmed that inoculation of *H. seropedicae* promotes higher accumulation of N in the biomass of maize (de Araujo et al., 2013; Alves et al., 2015; Breda et al., 2019; Dias et al., 2021). Interestingly, the greatest effects of the inoculation of this diazotrophic bacteria, especially in the metabolism of N, are reported under conditions of low availability of N. In addition, the application of the ZAE94 strain in maize seeds using peat as a carrier modified the plant response to N fertilization in a hybrid genotype, significantly increasing grain yield, mainly at the lowest tested dose of 0.8 mM of N (Alves et al., 2015). Similarly, our results corroborate these previous studies, as they demonstrate a beneficial effect of inoculation at a low-N dose of 0.3 mM, by increasing maize root and shoot biomass (**Figure 10**).

From the bacterial side, genes related to N fixation were also regulated in *H. seropedicae* during association with maize. In the INN+ vs CTN+ comparison, three *nif genes* were identified, encoding proteins involved in the synthesis, maturation and assembly of the nitrogenase complex (Chubatsu et al., 2012). *nifW* and *nifX* were significantly induced, while *fixU* had the expression repressed. Although the transcriptional control of N fixation and assimilation are well described in *H. seropedicae* (Chubatsu et al., 2012), N fixation during grass-bacteria associations is still poorly understood. The *nifH* and *nifA* gene expression profiles were up-regulated in both doses of N (0.3 mM and 3 mM) in the inoculated samples compared to the control, suggesting that the bacteria fixes N_2_ when associated with the plant. In addition, the expression levels of one of the components of the most studied two-component systems, the response regulators NtrC, and one of the main actors in the regulatory cascade that controls N metabolism in Proteobacteria, were up-regulated in plants inoculated under both doses of N.

The gene expression data showed that maize inoculation with the diazotrophic PGPB, *H. seropedicae*, reprogram both the plant and the bacteria N metabolism, with better responses in plants cultivated in low-N. It remains to be determined if the benefits observed to the plant are, at least in part, mediated by the direct transfer by the bacteria of the fixed N to the plant, or if it results from the ability of the PGPB to make N compounds available in the soil, to be assimilated by the plant.

### 4.2 Nitrogen-driven dynamics: orchestrating bacterial colonization and regulating plant defense hormonal pathways

Our findings may suggest that conditions of high N can have a negative effect on the stages of establishment and colonization by the bacteria, since the plant is in ideal growth conditions, developing independently of the beneficial association. In contrast, few DEGs were identified under low-N conditions, a regulatory profile that may be associated with a phase shift from bacterial colonization to an already well-established interaction (**Figure 10**).

During the establishment of the plant-bacteria interaction, the adhesion and host recognition stages are based on the release of signaling root exudates by the plants, which direct the free-swimming bacteria towards the roots (Balsanelli et al., 2016). In this study, three genes that encode LPS biosynthesis proteins had their expression reduced in INN+ vs CTN+. Previous studies have report that the attachment step of bacteria to the host roots is mediated by molecules of the bacterial cell envelope such as LPS, which actively contribute to early stages of the plant colonization process, being the first structures that contact the roots (Serrato et al., 2012; Balsanelli et al., 2016). In case of *H. seropedicae*, several studies reported that intact LPS is required for attachment and endophytic colonization of maize roots, since they are the outermost component of the outer membrane in Gram-negative bacteria (Balsanelli et al., 2010, 2013). The chemotaxis system acts through MCPs (Pereira et al., 2004). Two genes coding for MCPs, Hsero_1698 and Hsero_2164, had the expression increased in INN- vs CTN- comparison. Similar expression profiles of *MCP* genes were also observed in *H. seropedicae* SmR1 during the early stages of association with wheat roots (Pankievicz et al., 2016). The expression of several *MCPs* in *H. seropedicae* during maize colonization suggests that this endophytic bacterium could adapt its motility towards the host roots in response to different environmental signals, such as carbon sources, pH gradients and N availability (Balsanelli et al., 2016). Also, a gene involved in the flagellar formation, *flhF* was up-regulated in low N condition. Thus, our results indicate that, under low-N conditions, there is an increase in *MCP* gene expression in *H. seropedicae* ZAE94, suggesting that this induction has positive effects in bacterial motility and in the biosynthesis of flagella (**Figure 10**). In contrast, analysis of the INN+ vs CTN+ comparison revealed that *MCPs* genes were significantly repressed, and several genes that encode chemotaxis-related proteins were also down-regulated, suggesting that these experimental conditions may have a negative effect on the steps of establishment and colonization by bacteria (**Figure 10**). Similarly, repression of genes related to flagellar motility was also observed in INN+ vs CTN+ comparison, including down-regulation of flagellin gene *fliK*, as well as two genes involved in the formation of the flagellar basal body, *flgH* and *flgG*. The contrasting expression profile observed in response to the low-N and high-N conditions, could suggest that low N availability can generate a differential regulation, which induces the occurrence of colonization at an early stage in this case. The same expression profile was also described in *H. seropedicae* associated with several crops such as maize, wheat and sugarcane, a few days after inoculation (Balsanelli et al., 2016; Pankievicz et al., 2016; Pessoa et al., 2021).

Our results provided a complete panorama of gene expression of *H. seropedicae* during colonization of maize roots and revealed insights into pathways involved in a plant-bacteria interaction at later stages after inoculation (18 DAI). Notably, few DEGs involved in adhesion, chemotaxis, mobility, transport and secretion systems were identified under 0.3 mM of N conditions, a regulatory profile that may be associated with a change from initial bacterial colonization for an already well-established interaction (**Figure 10**). Our results corroborate the idea that the set of adhesion genes expressed depends possibly on the stage of colonization. Considering Roncato-Maccari et al. (2003) reported the presence of *H. seropedicae* occupying a xylem vessel of a maize stem by light microscopy, 15 DAI, it can be assumed that at this stage of later colonization, the bacteria have passed the adhesion phase and are located in host intercellular spaces. Moreover, although initial bacteria-plant communication may involve bacterial chemotaxis for root exudates, genes of the chemotaxis system were down-regulated under low-N conditions and at a later stage of colonization, indicating that this function became dispensable in the presence of plant fluid. Repression of genes related to chemotaxis and flagellar assembly were also reported in *H. seropedicae* HRC54 (Pessoa et al., 2021) and *Nitrospirillum amazonense* CBAmC (Terra et al., 2020) in response to sugarcane apoplastic fluid, suggesting that this plant fluid provides the nutrients necessary for bacteria metabolism, avoiding energy expenditure on bacterial motility. In addition, data obtained here suggest a transition from a mobile to a sessile lifestyle since genes associated with cell motility were not differentially regulated. Flavonoids can also influence the bacterial colonization process, and may modulate motility of *H. seropedicae* in maize rhizosphere (Tadra-Sfeir et al., 2011, 2015). Tadra-Sfeir et al. (2015) reported that, in the presence of the flavonoid naringerin in maize roots, flagella biosynthesis genes were highly repressed in *H. seropedicae* SmR1, in addition to regulating the expression of genes involved in the synthesis of the bacterial cell wall during the early stages of association with maize roots (Tadra-Sfeir et al., 2011). Since flavonoids represent a large fraction of root exudates involved in root colonization, the down-regulation of flagellar genes might also represent a phase shift in the colonization process, from free-swimming to sessile lifestyle by adhering to the maize roots (Pankievicz et al., 2016). These results corroborate data from scanning electron microscopy that report the detection of mucilage halos surrounding *H. seropedicae* in several areas on maize and sorghum roots, indicating heavy colonization 12 DAI (Roncato-Maccari et al., 2003). Our results suggest that at 18 DAI, at later stages of colonization, bacteria would have developed changes in their motility system to adapt to endophytic life by reducing flagella gene expression (**Figure 10**).

From the plant side, a differential regulation was observed in the expression of some genes involved in the biosynthesis and signaling pathways of SA, BR and ABA, phytohormones known to be involved in responses to biotic stresses, in response to the inoculation depending on the dose of N (**Figure 10**). An induction on the expression of some genes involved in the SA pathway in comparison INN+ vs CTN+, and a repression of these genes in other comparisons, suggested a better plant-bacteria association at low-N. SA is derived from phenolic compounds and is involved in the response to pathogen attacks (Vlot et al., 2009; An and Mou, 2011). *WRKY40* is a pathogen-induced transcription factor that was repressed in INN- vs CTN- and induced in INN+ vs CTN+. Several studies have described an important participation of salicylic acid in the regulation of endophytic colonization and diversity of endophytic bacterial populations (Pinski et al., 2019), as well as its essential role in the recruitment of specific rhizosphere microbiomes has been reported (Zhu et al., 2022). BR participates in the regulation of plant developmental processes, but also triggers responses to environmental stresses (Bajguz et al., 2020). Although the regulation of a specific pathway was not observed, we observed the negative regulation of CYP51, which acts decisively in the activation of defense against pathogens (Nelson and Werck-Reichhart, 2011), both in INN- vs CTN- and in CTN+ vs CTN-. Thus, we could infer an impairment of the plant’s basal defense response to beneficial inoculation. Some works have also demonstrated that the ABA pathway can be modulated by PGPB (Hardoim et al., 2019). Two genes involved in ABA biosynthesis were repressed CTN+ vs CTN-, but induced in the INN+ vs CTN+ comparison, *NCED9* and *AAO3* (Wang et al., 2016b), probably increasing ABA concentration in these plants. The repression of the ABA biosynthesis pathway observed in the CTN+ vs CTN- comparison demonstrates the need for N supplementation to ensure the growth of maize plants. Thus, the induction of this pathway observed in INN+ vs CTN+ may indicate that the combination of factors, inoculation and high dose of N, constitutes a stress for the plant by increasing ABA accumulation. In addition, *CYP707A* gene family plays a major regulatory role in controlling the level of ABA in plants, being involved in ABA catabolism (Kushiro et al., 2004). This gene was repressed in INN+ vs CTN+, suggesting an increase of ABA content in maize plants inoculated with *H. seropedicae* in 3 mM of N.

Taken together, our results demonstrate that the success of the plant-bacteria association depends on several factors, such as the perception of the nutritional status of the environment, which can trigger a more attenuated plant defense response in favorable conditions for this association; but also changes in bacterial metabolism through the specific regulation of genes involved in the plant-bacteria perception step (**Figure 10**).

### 4.3 Differential modulation of plant growth: integrating nitrogen levels with hormonal metabolism in plant-bacteria interactions

The experimental and environmental conditions applied in this work suggested that different doses of N fertilization differentially regulated the beneficial effects of bacterial inoculation. Modulation of plant hormones by PGPB can increase plant nutrient uptake and regulate stress responses, promoting plant growth (Glick, 2012). Also, most PGPB synthetize plant growth hormones. *A. brasilense* Ab-V5 produces IAA and has strong plant growth-promoting capacity, leading to increase in auxin concentration, which possibly favors plant biomass and a change in root architecture (Zeffa et al., 2019). *H. seropedicae* produces auxin (IAA) and gibberellin (GA), implicated in various processes of plant growth and development (Irineu et al., 2022). Our work identified that Hsero_4278, which encodes the enzyme indolepyruvate ferredoxin oxidoreductase responsible for the conversion of indolepyruvate into IAA, was up-regulated in both N doses, but showed more pronounced levels in the comparison INN+ vs CTN+, suggesting that, after 18 DAI, the bacteria may be producing IAA under both N availability, but mainly under conditions of increased N availability.

From the plant side, our results showed that most of the hormone pathways have the same regulation in both CTN+ vs CTN- and INN- vs CTN- comparisons and are opposite to the regulation observed for INN+ vs CTN+ (**Figure 10**). This result suggests that inoculation with the bacteria at low-N could share similar positive effects in promoting plant growth and modulating hormonal pathways to those observed with high N fertilization. Our analyses suggested that auxin biosynthesis could be up-regulated in inoculated maize roots, cultivated with 3 mM of N. In the auxin signaling, the *Arabidopsis* ARF2 and ARF3 transcription factors are repressors of expression of auxin responsive genes (Finet et al., 2013). In inoculated maize plants, *ARF2* was repressed in the comparison INN+ vs CTN+, while *ARF3* was down-regulated in low-N (INN- vs CTN-). *ARF2* has previously been reported to be also repressed in rice in response to inoculation by *H. seropedicae* (Brusamarello-Santos et al., 2012). Furthermore, a global induction of auxin-responsive genes such as *GLP4*, *FRQ1* and *AVP1* were identified in the INN+ vs CTN+ comparison, suggesting that under elevated N conditions bacteria could be contributing to promotion of maize growth. In *Arabidopsis*, overexpression of *AVP1* increased auxin transport, resulting in robust root development (Li et al., 2005). These results suggest that inoculation of *H. seropedicae* can affect the repression of specific genes of the auxin signaling pathway that could be involved in the promotion of root growth. In addition, genetic repression of members of the auxin pathway may also be important for the bacteria inside the plant, negatively regulating the defense system. Further studies are necessary to elucidate whether and how the auxin pathway participates in plant-bacteria interactions.

Gibberellins can also promote cell elongation and division (Sauter and Kende, 1992). It is important to highlight that the gibberellin produced by PGPB could also contribute to increasing root hair density (Bottini et al., 2004). Hormonal quantification showed a higher content of the GA1 and GA3 forms of gibberellin in inoculated maize roots, while the maize genes involved in the GA biosynthesis appear to be almost all repressed (Irineu et al., 2022). This result suggested that the production of gibberellin occurred through the bacterial activity of *H. seropedicae*. However, in our results, we observed that *GA1*, *GA2* and *GA3* were induced in the comparison INN+ vs CTN+, suggesting that bacterial inoculation activated plant GA synthesis. Remarkably, at higher N concentrations, *H. seropedicae* promoted other benefits to the plant, such as shoot length growth, without gain of dry mass (**Figure 9**). *EXPA1* was also up-regulated in INN+ vs CTN+, suggesting changes in the chemical composition of the cell wall that could be possibly modulating root development in response to inoculation under N+, as observed in the overexpression of *EXPA1* in Arabidopsis mutant seedlings (Samalova et al., 2023). Thus, our molecular and phenotypic results suggested that under conditions of high N availability, and in the presence of *H. seropedicae*, there is growth promotion in maize plants, without gain of dry mass. Further cellular and biochemical experiments in plants inoculated in high and low-N are necessary to elucidate mechanisms involved in the differential biomass acquisition by bacterial inoculation in these two conditions, at the stage of plant-bacteria association analyzed in our experiments.

Cell walls constitute more than 50% of the weight of dry biomass, which is largely dependent on cell wall components (López-Malvar et al., 2022), being candidates to be contributing, at least in part, to the different phenotypes observed in plants inoculated in low and high-N (Ballesteros et al., 2021). In contrast to the patterns observed in N metabolism and the hormonal pathway, the set of DE genes in the different cell wall-related families did not show a similar regulation of genes between CTN+ vs CTN- and INN- vs CTN- comparisons (**Figure 10**). Cellulose synthase and cellulose-like synthase genes related to primary and secondary wall formation can be differentially expressed according to tissue type (Cosgrove, 2005). Isoforms of the genes *ZmCESA1* to *ZmCESA9* are involved in the primary cell wall synthesis, while the genes *ZmCESA10* to *ZmCESA12*, and their isoforms, are associated with secondary cell wall biosynthesis (Penning et al., 2019; Kozlova et al., 2020). Our study showed that the *CESA9* gene is induced in plants inoculated with *H. seropedicae* at N+ concentrations, suggesting that the presence of bacteria in this condition may regulate the expression of genes related to greater deposition of cellulose in the root cell walls. On the other hand, the *CESA* genes (*CESA4, CESA7* and *CESA8*), involved in the secondary wall formation, were mainly induced in plants inoculated with *H. seropedicae* cultivated under low-N. In *A. thaliana*, *cesa4^irx5-4^*, *cesa7^irx3-7^*, and *cesa8^irx1-7^* mutants exhibit dark green leaves and inflorescence stems; reduced plant height, lower cellulose content and collapsed xylem (Kumar et al., 2017). Thus, our data suggests that the presence of bacteria in low-N condition may promote a greater deposition of cellulose in the root primary cell walls, leading to increase in biomass gain. The regulation of *CESA* genes through the association with beneficial bacteria was also observed in contrasting genotypes for BNF in sugarcane plants (Ballesteros et al., 2021). The genotype best associated with the PGPB showed regulation of genes involved in primary and secondary cell walls, in response to bacterial association, increasing the cellulose contents and non-cellulolytic components in the cell walls of root cells.

In addition, several genes correlated with root growth and the vascular pattern of the cotyledons were differently regulated, including *SKU5* gene which encodes a family of multi-copper oxidase-like proteins with cupredoxin domains similar to laccase and ascorbate oxidase (Zhang et al., 2021). Our data showed that the *SKS12* and *SKS13* genes were up-regulated in plants inoculated with *H. seropedicae* cultivated in high and low-N conditions, respectively, indicating that the bacteria would have an effect on the regulation of these genes, and that it could induce the formation of lateral roots and root elongation as shown in other studies (Sedbrook et al., 2002). The *sku5* mutant exhibits a phenotype of skewed and looped roots, suggesting that *SKU5* is required for directional root growth, possibly via an effect on cell expansion (Sedbrook et al., 2002). Therefore, our findings showed that the bacteria induce different forms of growth depending on the nutritional availability of the soil in relation to the N concentration, either lengthening as seen in high N or increasing biomass as seen in low-N.

Several evidences suggest that PGPB can regulate the N metabolism of grasses, or promote greater efficiency in the use of N, contributing to the status of this nutrient in the plant (Carvalho et al., 2014; Breda et al., 2019; Zeffa et al., 2019). Our results provided the first integrated overview of differentially expressed genes in both maize and associated *H. seropedicae* ZAE94 in response to different N availability, revealing intricate biochemical and physiological mechanisms governing beneficial plant-microbe interactions. While existing studies have focused on early plant recognition and colonization stages by *H. seropedicae*, our research sheds light on the transcriptional profile of *H. seropedicae* during the later stages of the association when the plant-bacteria interaction is well established. Taken together, our results demonstrate that the efficiency of a plant-bacteria association depends on several factors, such as the perception of the nutritional status of the environment and changes in plant and bacterial metabolism, which can form a combined response, leading to a more improved association and ensuring the promotion of plant growth and adaptation (**Figure 10**). The data provide a valuable contribution to how different N doses can alter the metabolism of maize and *H. seropedicae*. Our results corroborate several studies that propose that PGPB inoculation can lead to an increase in NUE, improving nutrient absorption and increasing dry matter, reducing dependence on synthetic N fertilizers. A further understanding of the relationship between plant-bacteria-environment signaling could potentially offer sustainable alternatives to reduce chemical fertilizers dependence in agriculture.

## Data availability statement

The original contributions presented in the study are included in the article/**Supplementary Files**, further inquiries can be directed to the corresponding author/s.

## Author contributions

AR: Data curation, Investigation, Methodology, Writing – original draft, Writing – review & editing. MU: Data curation, Methodology, Validation, Writing – original draft, Writing – review & editing. FT: Writing – original draft. HF: Writing – original draft. EO: Writing – original draft. AH: Funding acquisition, Supervision, Writing – review & editing.
