# Supplementary figures and images for: Dual RNA-seq of maize and H. seropedicae ZAE94 association, in different doses of nitrate, reveals novel insights into Plant-PGPB-environment relationship
Source: Front Plant Sci. 2024 Mar 13;15:1346523. doi: 10.3389/fpls.2024.1346523 (PMC10965572; doi:10.3389/fpls.2024.1346523)

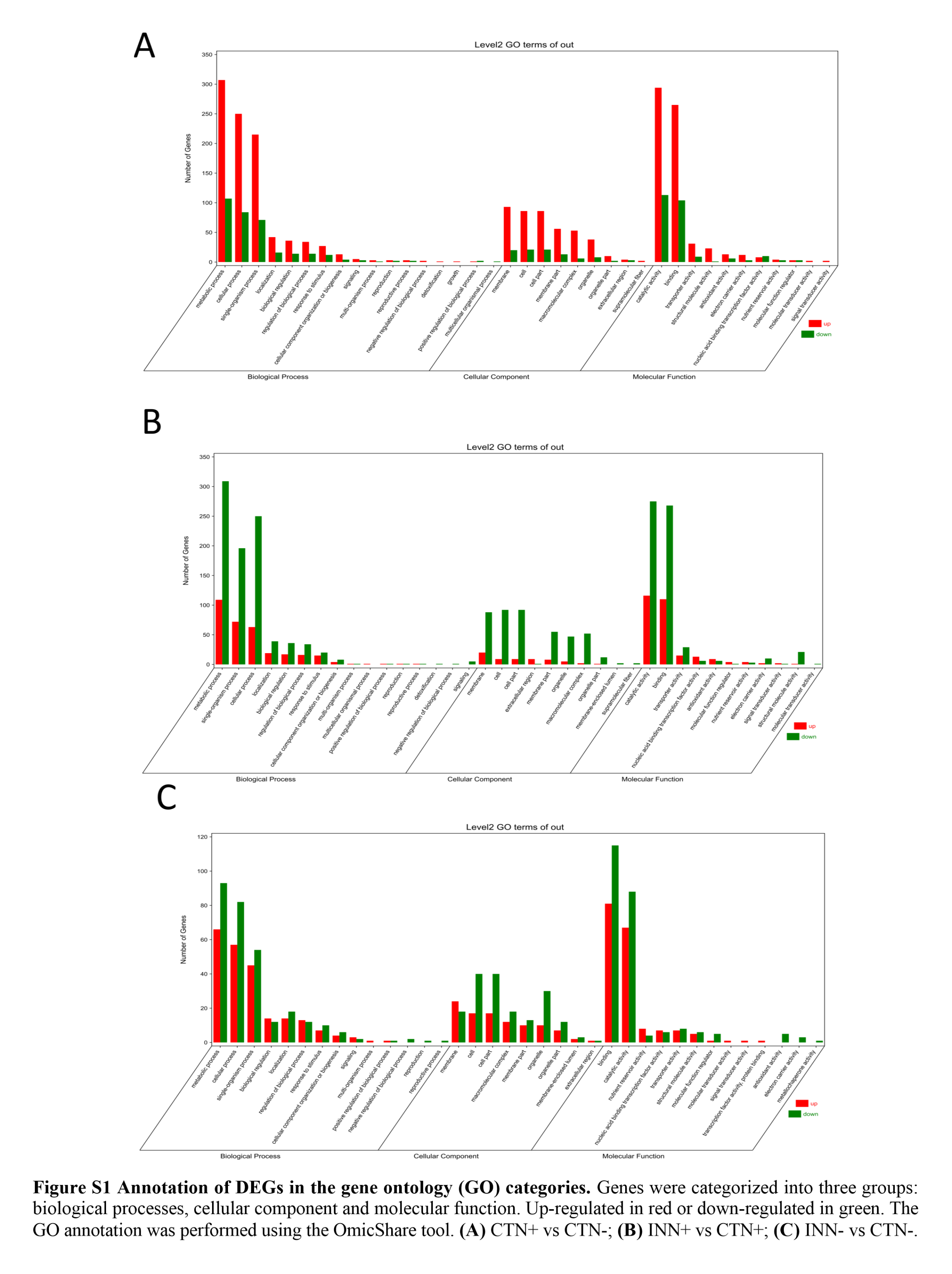

Supplement: Supplementary file 1 [file Image_1.tif]

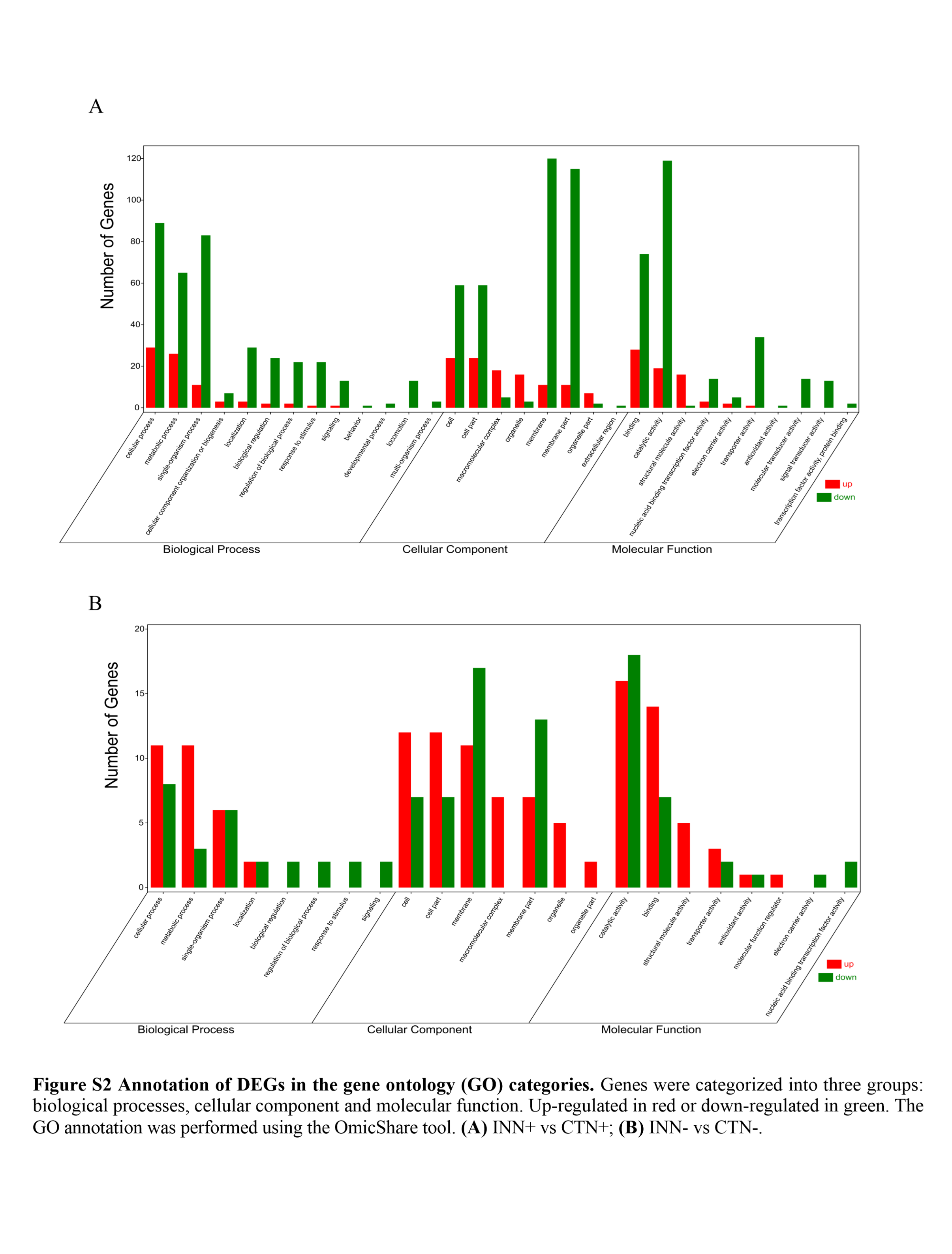

Supplement: Supplementary file 2 [file Image_2.tif]

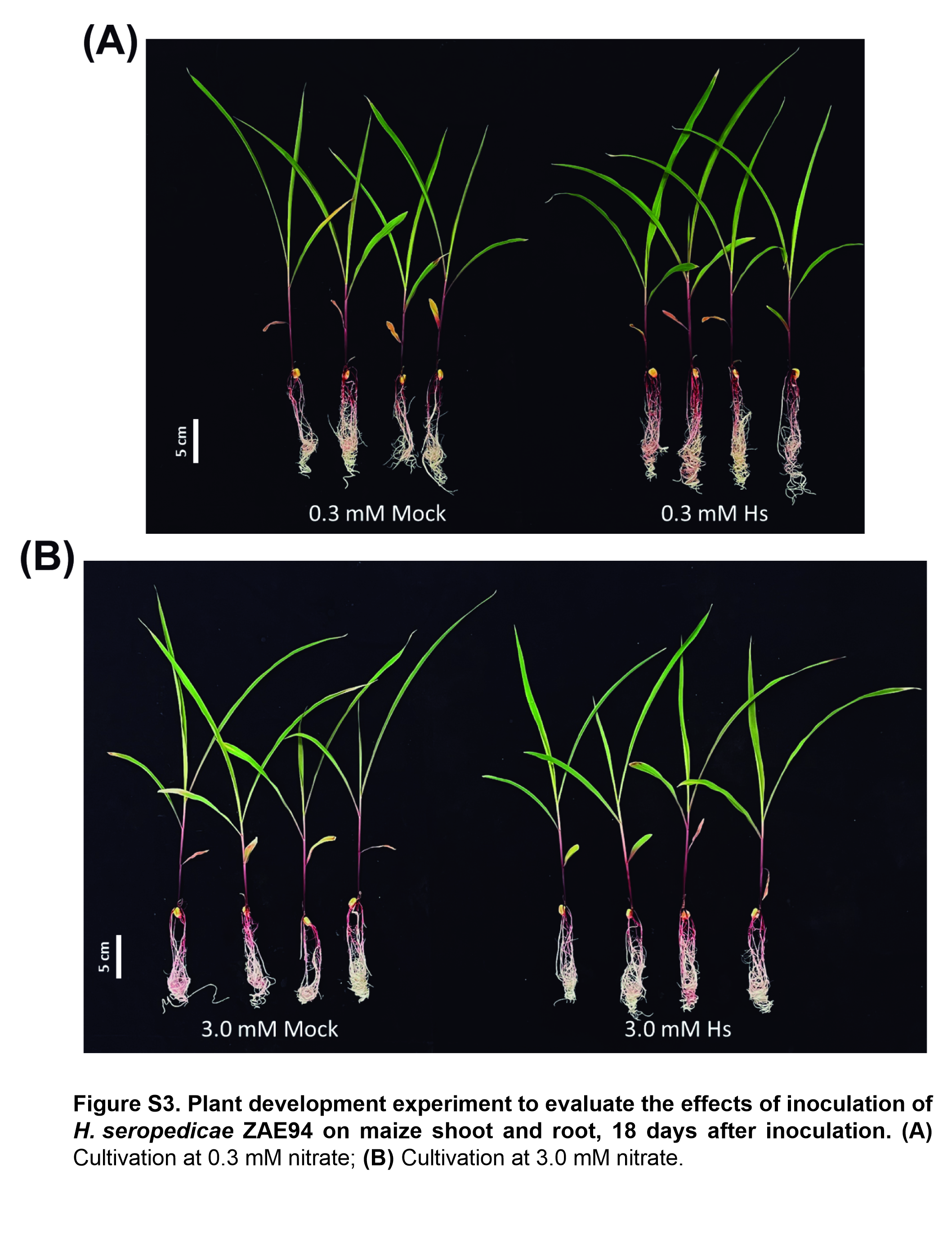

Supplement: Supplementary file 3 [file Image_3.tif]
