# Supplementary material for: Dual RNA-seq of maize and H. seropedicae ZAE94 association, in different doses of nitrate, reveals novel insights into Plant-PGPB-environment relationship
Source: Front Plant Sci. 2024 Mar 13;15:1346523. doi: 10.3389/fpls.2024.1346523 (PMC10965572; doi:10.3389/fpls.2024.1346523)
Supplement: Supplementary file 4 [file Table_1.docx]

**Table S1** Composition of Hoagland solution.

**Hoagland composition**

| **Nutrient** | **0.3 mM (mg L^-1^)** | **1.5 mM (mg L^-1^)** | **3.0 mM (mg L^-1^)** |
| --- | --- | --- | --- |
| N-NO_3_^-^ | 3.5 | 17.5 | 35 |
| N-NH_4_^+^ | 0.7 | 3.5 | 7 |
| P | 15.5 | 15.5 | 15.5 |
| K | 117 | 117 | 117 |
| S-SO_4_^-^ | 123.38 | 110.5 | 94.62 |
| Ca | 80 | 80 | 80 |
| Mg | 24 | 24 | 24 |
| Fe | 3 | 3 | 3 |
| B | 0.5 | 0.5 | 0.5 |
| Cu | 0.02 | 0.02 | 0.02 |
| Mn | 0.6 | 0.6 | 0.6 |
| Zn | 0.05 | 0.05 | 0.05 |
| Mo | 0.08 | 0.08 | 0.08 |
