# Supplementary material for: Dual RNA-seq of maize and H. seropedicae ZAE94 association, in different doses of nitrate, reveals novel insights into Plant-PGPB-environment relationship
Source: Front Plant Sci. 2024 Mar 13;15:1346523. doi: 10.3389/fpls.2024.1346523 (PMC10965572; doi:10.3389/fpls.2024.1346523)
Supplement: Supplementary file 5 [file Table_2.docx]

**Table S2** Summary of the RNA-seq library trimming and mapping statistics. Maize plants were inoculated with *Herbaspirillum seropedicae* ZAE94 (IN) or mock-inoculated (CT) and cultivated in 0.3 mM (N-) or 3 mM of nitrate. High quality reads were aligned to B73 reference genome (ZmB73_RefGen_v4) and *H. seropedicae* Z67 (NZ_CP011930.1).

**Reads and mapping stats for *Zea mays***

| Sample | Sample name | No. Reads (R1+R2) | No. mapped reads | %mapped  reads | secondary  mappings | %multiple  mappings |
| --- | --- | --- | --- | --- | --- | --- |
| CTN- | ADSF3 | 54904078 | 3138901 | 5.72% | 11'516'600 | 86.91% |
|  | FAS-2338 | 62940808 | 6528925 | 10.37% | 18'589'180 | 82.82% |
| CTN+ | ADSF4 | 60142696 | 39246681 | 65.26% | 130'056'536 | 86.26% |
|  | FAS-2341 | 59213944 | 3024781 | 5.11% | 8'268'074 | 81.80% |
| INN- | ADSF1 | 66214136 | 5960118 | 9.00% | 19'252'120 | 84.80% |
|  | FAS-2339 | 66363336 | 10535847 | 15.88% | 35'312'166 | 85.68% |
| INN+ | ADSF2 | 67316920 | 7046524 | 10.47% | 25'825'975 | 86.92% |
|  | FAS-2340 | 58552000 | 7464957 | 12.75% | 23'010'773 | 84.06% |

**Reads and mapping stats for *H. seropedicae***

| Sample | Sample name | No. Reads (R1+R2) | No. Mapped reads | %mapped  reads | secondary  mappings | %multiple mappings |
| --- | --- | --- | --- | --- | --- | --- |
| CTN- | ADSF3 | 54904078 | 4936618 | 8.99% | 3'858 | 3.75% |
|  | FAS-2338 | 62940808 | 3462164 | 5.50% | 3'288 | 3.43% |
| CTN+ | ADSF4 | 60142696 | 363643 | 0.60% | 56 | 7.95% |
|  | FAS-2341 | 59213944 | 3658850 | 6.18% | 3'714 | 3.12% |
| INN- | ADSF1 | 66214136 | 5790454 | 8.75% | 3'984 | 4.15% |
|  | FAS-2339 | 66363336 | 3172414 | 4.78% | 3'480 | 4.14% |
| INN+ | ADSF2 | 67316920 | 6567623 | 9.76% | 5'938 | 3.64% |
|  | FAS-2340 | 58552000 | 3476350 | 5.94% | 3'600 | 3.16% |
